# Enteric bacterial infection stimulates remodelling of bile metabolites to promote intestinal homeostasis

**DOI:** 10.1038/s41564-024-01862-z

**Published:** 2024-11-20

**Authors:** Ting Zhang, Yuko Hasegawa, Matthew K. Waldor

**Affiliations:** 1 Division of Infectious Diseases, Brigham and Women’s Hospital, Boston, MA USA; 2 Department of Microbiology, Harvard Medical School, Boston, MA USA; 3 Howard Hughes Medical Institute, Boston, MA USA

**Keywords:** Pathogens, Bacterial host response

## Abstract

The liver makes bile, an aqueous solution critical for fat absorption, which is secreted into the duodenum. Despite extensive studies on bile salts, other components of bile are less well characterized. Here we used global metabolomic analysis on bile from specific-pathogen-free, germ-free, *Citrobacter rodentium*-infected or *Listeria monocytogenes*-infected mice and identified a metabolome of 812 metabolites that were altered by both microbiota and enteric infection. Hepatic transcriptomics identified enteric-infection-triggered pathways that probably underlie bile remodelling. Enteric infection increased levels of four dicarboxylates in bile, including itaconate. Analysis of *Acod1*^−/−^ mice indicated that increased itaconate also increased tuft cell abundance, altered microbiota composition and function as detected by metagenomic analysis, and modulated host defence, leading to reduced *Vibrio cholerae* colonization. Our data suggest that enteric-infection-associated signals are relayed between the intestine and liver and induce transcriptional programmes that shape the bile metabolome, modifying the immunomodulatory and host defence functions of bile.

## Abstract (web summary)

Metabolomic analysis of bile after infection by enteric bacteria in mice reveals composition changes including increased itaconate levels that promote intestinal homeostasis and reduce *Vibrio cholerae* colonization.

## Main

**Fig. 1:**
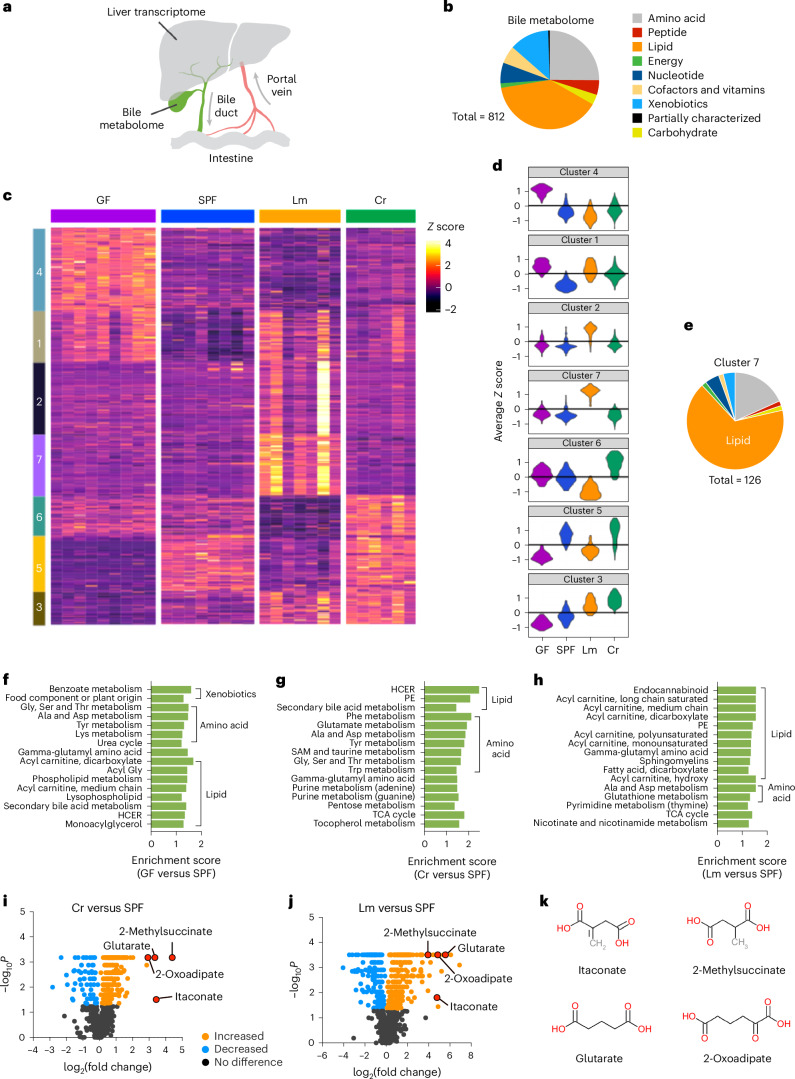
The microbiota and enteric infection modify the bile metabolome. **a**, Schematic of enterohepatic circulation and experimental design. The liver receives blood from the portal vein that drains the intestine and produces bile that is delivered to the duodenum via the common bile duct. **b**, Functional categories of the 812 bile metabolites in SPF mice that were identified by global metabolomic profiling. Energy category refers to metabolites within the citric acid cycle. Detailed subsets of lipid molecules are shown in Fig. 2. Molecules in each functional category are shown in Supplementary Table 1b. **c**, Unsupervised clustering (*k*-medoids) of the bile metabolites identified in four groups of mice (GF (*n* = 9), SPF (*n* = 8), Lm (*n* = 7) and Cr (*n* = 6)) based on their patterns of relative abundance. Each row represents a metabolite and each column represents one sample. To obtain a minimal volume of 60 µl of bile for global metabolomic profiling, samples were generated by pooling bile from 3 to 5 SPF mice, 3 or 4 Lm or Cr mice, and 2 or 3 GF mice; a total of 146 mice were used. **d**, Violin plots of the distribution of the *Z* scores of the metabolites in each cluster. **e**, Functional categories of bile metabolites in cluster 7 identified by unsupervised clustering. **f**–**h**, Enriched pathways of metabolites with differential abundance between SPF and GF mice (**f**), SPF and Cr mice (**g**) and SPF and Lm mice (**h**). PE, phosphatidylethanolamine; SAM, S-Adenosyl methionine; HCER, hexosylceramides. Pathways with the top 16 enrichment scores are listed for each comparison. **i**,**j**, Differential abundance of bile metabolites in Cr (**i**) or Lm (**j**) animals compared with that in uninfected mice. Blue, black and orange dots represent metabolites with reduced, unchanged or increased abundance, respectively, in the samples from infected animals. *P* values were generated using the two-tailed Wilcoxon rank test. **k**, Structural formulas of infection-stimulated bile dicarboxylates. Source data

The cross-kingdom interplay between microbiomes and their mammalian hosts generates a diverse pool of compounds, many of which enter the circulatory system and impact host organ function^1–3^. The liver receives blood from the portal vein, which drains the intestine, and from the systemic circulation, allowing it to play a critical role in integrating chemical signals from the diet and the gut microbiome with those present in systemic blood (Fig. 1a). The liver responds to these stimuli by synthesizing a large array of compounds that are secreted into the systemic circulation and modulate host physiology. In addition, infection or tissue injury can trigger the ‘acute phase response’, in which hepatocytes produce and secrete large amounts of proteinaceous mediators into the systemic circulation, which facilitate host defence and/or repair at distal sites^4,5^.

Besides synthesizing many of the noncellular components of blood, the liver also plays a central role in the formation of bile, an aqueous solution that can be stored in the gall bladder before its transport to the duodenum via the common bile duct (Fig. 1a)^6^. Bile consists of lipids, proteins, metabolites and bile acids^7^. Although the functions of bile acids have received considerable attention^8–10^, the chemical composition, functions and regulation of other bile constituents have not been the subject of much experimental scrutiny.

In contrast to the widely surveyed serum metabolome^1,3^, changes in bile composition in response to the microbiota or enteric infection are largely unknown. As bile is a key mediator of interorgan communication between the liver and gut, we hypothesized that enteric infection may alter the composition and function of bile to facilitate intestinal defence. Here we used global metabolomic analyses to characterize mouse bile and to investigate how its composition is altered by microbiota and enteric infection. Our findings reveal that bile composition is highly complex, responsive to the microbiota and infection, and functions in an interorgan innate defence circuit that links the liver and intestine.

## Results

### Profiling the bile metabolome

**Fig. 2:**
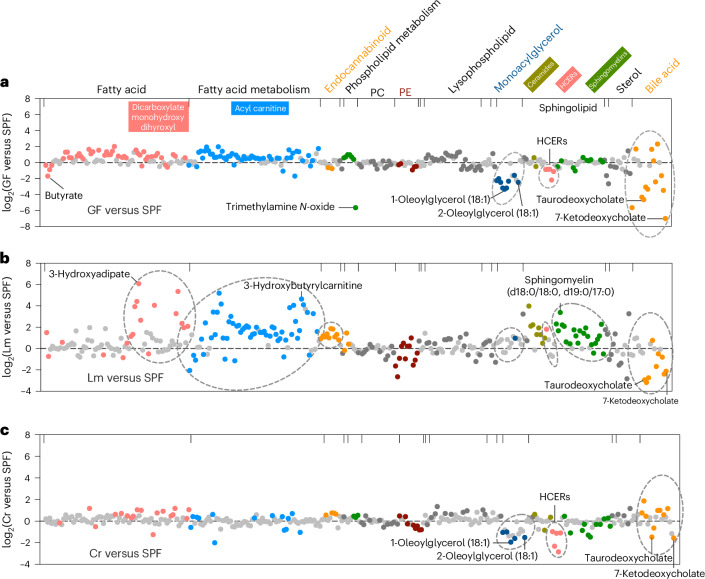
The bile lipidome is shaped by the microbiota and modified by enteric infection. **a**–**c**, Differential abundance of bile lipids in GF (**a**), Lm (**b**) or Cr mice (**c**) compared with SPF mice. Each dot represents one lipid; the categories of lipids are found at the top of the graph in **a**. PC, phosphatidylcholine; PE, phosphatidylethanolamine. The *y*-axis represents the log_2_ fold change of the metabolite abundances in the experimental conditions (GF, Lm and Cr in the numerator) relative to the SPF condition (denominator). *P* values were generated using the two-tailed Wilcoxon rank test. Light grey dots indicate that statistically significant difference was not reached; colored dots indicate that statistically significant difference was reached (*P* < 0.05). Lipid sub-pathways with high enrichment scores by pathway enrichment analysis are indicated with dashed circles. Source data

**Extended Data Fig. 1:**
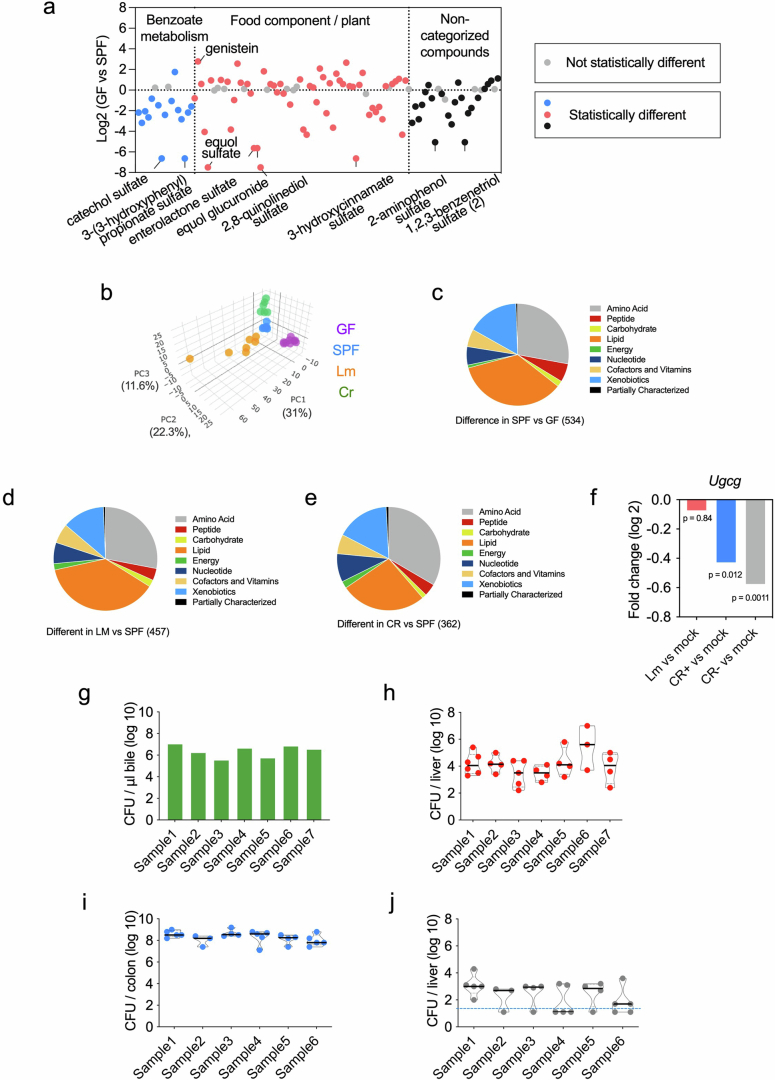
Microbiota and enteric infection alter the bile metabolome. (**a**) The relative abundance of metabolites in benzoate metabolism (blue), food components (pink), and non-categorized compounds (black) in bile of GF mice compared to SPF mice. p values were generated using two-tailed Wilcoxon rank test. Dots labeled in light-grey do not reach statistical difference; dots labeled in other colors are statistically different (p<0.05). (**b**) Principal component analysis (PCA) of the bile metabolome in animals from the following four groups of mice: SPF, specific pathogen free, GF, germ free, Lm, *L. monocytogenes* infected, and Cr, *C. rodentium* infected. Each dot represents one sample; percentage represents fraction of variance explained by each principal component. (**c–e**) The functional categories of metabolites with differential abundance in the indicated comparison. (**f**) The relative expression level of *Ugcg* in mouse liver in Lm vs SPF, Cr liver positive vs SPF, and Cr liver negative vs SPF conditions. Adjusted p values were generated using DESeq2 (two-tailed Wald test p-value, adjusted for multiple testing using the procedure of Benjamini and Hochberg). (**g–j**) The CFU of *L. monocytogenes* in bile from the gallbladder (**g**) and liver (**h**) at 4-day post orogastric infection, and *C. rodentium* in colon (**i**) and liver (**j**) at 10-day post orogastric infection. The dotted line represents the limit of detection. Source data

**Extended Data Fig. 2:**
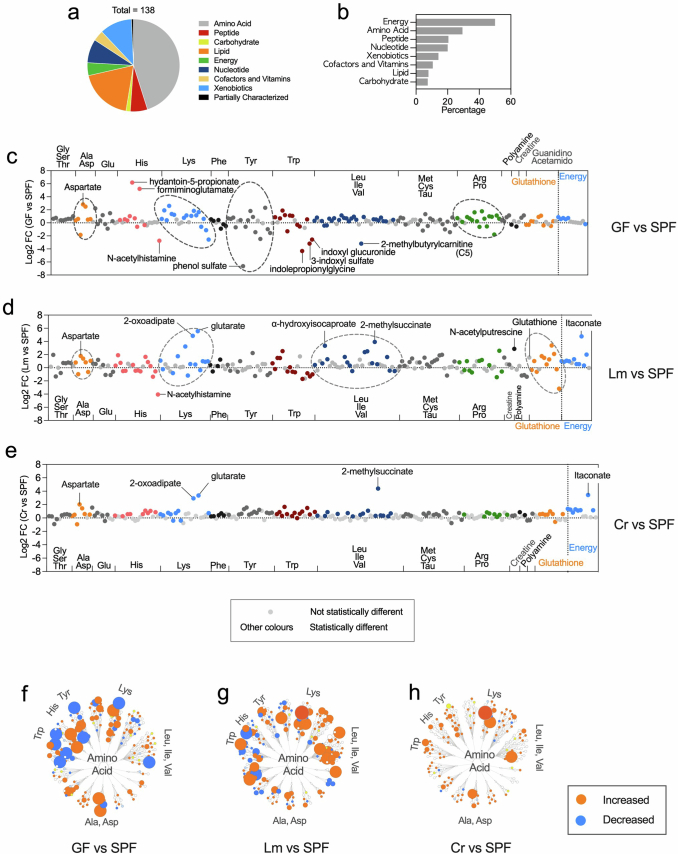
Microbiota and enteric infections alter the abundance of bile metabolites related to amino acids and energy. (**a**) The functional categories of bile metabolites that were elevated in both Lm and Cr infection. (**b**) The percentage of metabolites in each functional category that were elevated in both infection conditions. (**c–e**) The differential abundance of metabolites in the indicated categories. Comparisons of GF vs SPF (**c**), Lm vs SPF (**d**), and Cr vs SPF (**e**) are shown. p values were generated using two-tailed Wilcoxon rank test. Dots labeled in light-grey do not reach statistical difference; dots labelled in other colours are statistically different (p < 0.05). (**f**-**h**) Changes in abundance of metabolites linked to amino acid metabolism pathways in GF vs SPF (**f**), Lm vs SPF (**g**), Cr vs SPF (**h**). Dots labelled in blue and orange were statistically significant and represent decreased and increased abundance respectively; the size of the dots varies according to the magnitude of the differ. Source data

To expand understanding of the chemical diversity of mouse bile, we used global metabolomic analysis to profile mouse bile metabolites. Bile samples were obtained from the gall bladders of C57BL/6 specific-pathogen-free (SPF) animals. In total, 812 metabolites, representing 9 functional categories, were identified in the bile (Fig. 1b and Supplementary Table 1a,b). Lipids are the dominant class of bile components (Fig. 1b) and include diverse subsets of molecules (Fig. 2). In addition, several metabolites representing intermediate products in amino acid and nucleotide metabolism were also identified (Supplementary Table 1c). Bile metabolites were not only of host origin. Many of the compounds in the bile of SPF mice are generated or processed by the gut microbiota and have been identified in the host portal and systemic circulation (for example, equol sulfate and indoxyl sulfate)^11,12^ (Extended Data Figs. 1a and 2c, and Supplementary Table 1d). Furthermore, several bile constituents, such as hydroxycinnamate and genistein, are of dietary origin (Extended Data Fig. 1a and Supplementary Table 1e). The presence of microbial- and dietary-derived compounds in bile suggests that they are distributed through enterohepatic circulation and, following their intestinal absorption, processed by the liver and secreted in bile, similar to xenobiotics^11^.

To investigate how the microbiota and enteric infection impact bile composition, we compared the metabolomes of bile from SPF mice with those from germ-free (GF) animals, and mice orally infected with *Listeria monocytogenes* (Lm) or *Citrobacter rodentium* (Cr). An unsupervised clustering algorithm was used to classify the bile metabolites identified in the four conditions based on their patterns of abundance. The 812 bile metabolites were partitioned into 7 clusters, which in aggregate distinguished the conditions (Fig. 1c,d and Supplementary Table 2). Principal component analysis and pathway enrichment analysis also revealed that the bile metabolomes in these four conditions were distinct (Fig. 1f–h, Extended Data Fig. 1b and Supplementary Table 3).

### Bile metabolites are shaped by the microbiota

The makeup of bile in SPF and GF mice was easily distinguishable (clusters 4, 1, 5 and 3; Fig. 1c,d). The relative abundance of nearly 60% of the metabolites differed between the two groups (*P* < 0.05; Extended Data Fig. 1c). As expected, many compounds that are generated or processed by the gut microbiota (for example, indolepropionylglycine) were found in the bile of SPF and not GF animals (Extended Data Fig. 2c). Compounds classified as xenobiotics, amino acids and lipids were the most affected by the absence of the microbiota (Fig. 1f).

The bile lipidome was also distinct in GF mice. As expected, known microbial-derived lipids such as secondary bile acids and short-chain fatty acids had reduced abundance in GF versus SPF animals (Fig. 2a). The absence of the microbiota was also associated with reduced abundance in bile of several monoacylglycerols (for example, 1-oleoylglycerol (18:1)) and hexosylceramide (HCER; Fig. 2a), suggesting that the microbiota contributes to the production or metabolism of these lipids. These observations, together with previous studies^13–16^, reinforce the idea that the interplay between the microbiota and host has a profound impact on bile composition.

### Enteric infection modifies bile composition

We hypothesized that enteric infection alters bile composition because infection probably stimulates changes in the composition of portal blood owing to the delivery of microbiota- and pathogen-derived molecules and/or host-intestine-derived signalling molecules such as cytokines to portal circulation. The profiling of bile metabolites was carried out 4 and 10 days following oral inoculation of mice with *L. monocytogenes* and *C. rodentium*, respectively. *L. monocytogenes* routinely disseminates from the intestine to the liver and gall bladder following oral inoculation^17^. By contrast, *C. rodentium*, a natural mouse enteric pathogen, primarily replicates in the caecum and colon. Four days postinfection (dpi), the burden of *L. monocytogenes* in the liver and the gall bladder peaks^18^; in the latter organ, the pathogen replicates extracellularly in bile where it reaches concentrations of ~10^9^ colony forming unit (CFU) ml^−1^ (Extended Data Fig. 1g,h). *C. rodentium* was never isolated from the gall bladder, and a peak pathogen burden of ~10^9^ CFU was observed in the colon at 10 dpi (Extended Data Fig. 1i). At this point, in some animals, a small number (median, 400) of *C. rodentium* CFU were isolated from the liver (Extended Data Fig. 1j). Both pathogens altered bile metabolite profiles compared with SPF animals, although the changes of the metabolites differed between pathogens as represented in the clustering results (Fig. 1c,d,g,h and Extended Data Fig. 1d,e). Thus, even in the absence of pathogen invasion of the gall bladder, enteric infection leads to remodelling of the bile metabolome in a pathogen-specific manner.

*L. monocytogenes* infection altered the abundance of more bile metabolites (457) than *C. rodentium* infection (362; Fig. 1c, Extended Data Fig. 1d,e and Supplementary Tables 1b and 2). Metabolites in cluster 2 and 7, which were highly enriched for lipids (Fig. 1e and Supplementary Table 2), were only heightened in *L. monocytogenes*-infected animals (Fig. 1c,d). Pathway enrichment analysis of all differentially abundant metabolites also revealed that *L. monocytogenes* infection triggered the alteration of the bile lipidome (Figs. 1h and 2b, and Extended Data Fig. 1d). In contrast to bile from the GF, SPF and Cr groups of animals, bile from the Lm group had elevated abundances of fatty acids, acyl-carnitines, endocannabinoids and sphingomyelins (Fig. 2b), potentially in part owing to the activities of the pathogen’s phospholipases^19^. By contrast, *C. rodentium*-infected animals, like GF mice, had decreased abundance of HCERs and monoacylglycerols (Fig. 2a,c), suggesting that alterations in the microbiota caused by the proliferation of *C. rodentium* in the intestine^20^ reduced the abundance of these likely microbiota-derived bile lipids. In addition, as HCERs can also be generated by de novo synthesis in mammalian tissues by the enzyme UDP-glucose ceramide glucosyltransferase (UGCG)^21^, an alternative explanation is that *C. rodentium* suppresses the activity of UGCG. Indeed, we found that infection by *C. rodentium*, not by *L. monocytogenes*, downregulates hepatic *UGCG* expression (Extended Data Fig. 1f), supporting the idea that *C. rodentium* replication in the intestine can impact the expression of this host lipid-synthesizing enzyme.

**Extended Data Fig. 3:**
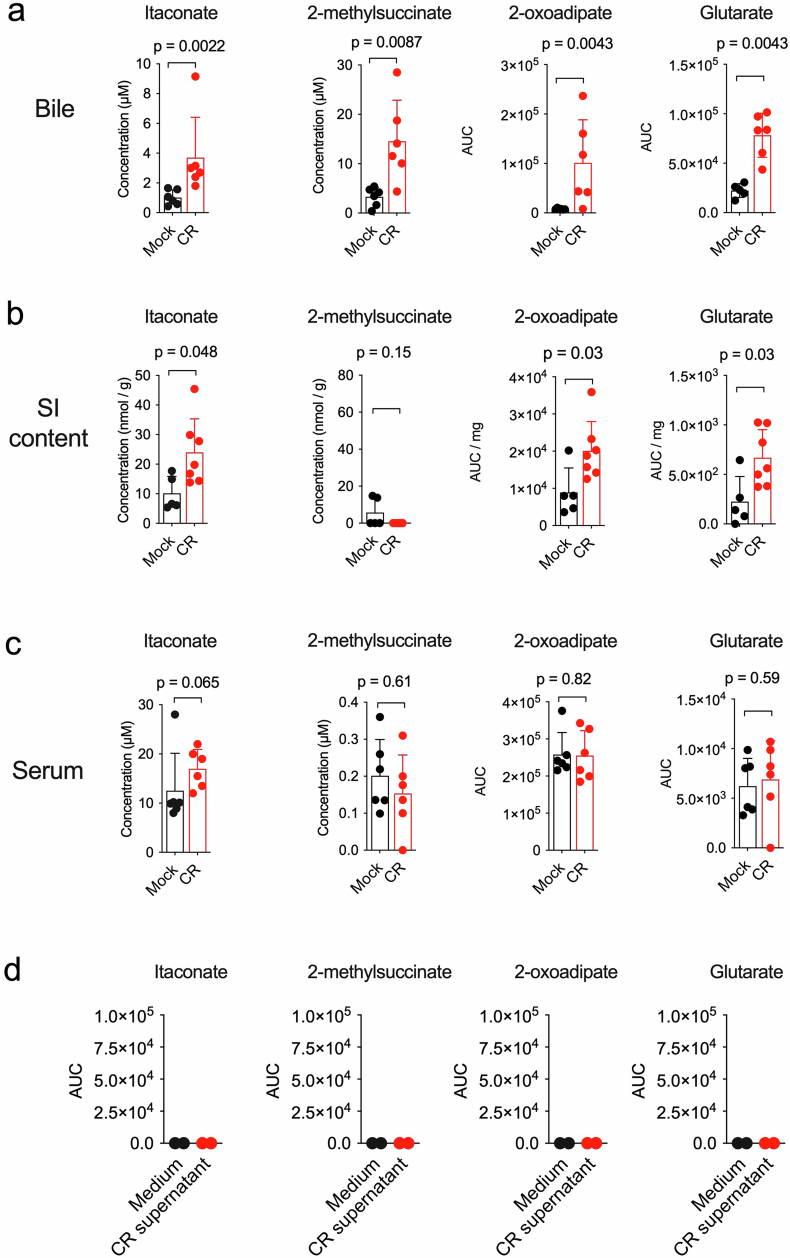
Abundance of itaconate, 2-methylsuccinate, glutarate, and 2-oxoadipate in tissues and bacterial culture. (**a**) The absolute abundance of itaconate and 2-methylsuccinate, and relative abundance of 2-oxoadipate in bile of uninfected animals (n = 6) and Cr infected mice (n = 6), and relative abundance of glutarate in bile of uninfected animals (n = 5) and Cr infected mice (n = 6). Data are presented as mean values +/− SD. (**b**) The absolute abundance of itaconate and 2-methylsuccinate, and relative abundance of glutarate and 2-oxoadipate in proximal small intestine contents of uninfected animals (n = 5) and Cr infected mice (n = 7). Data are presented as mean values +/− SD. (**c**) The absolute abundance of itaconate and 2-methylsuccinate, and relative abundance of glutarate and 2-oxoadipate in serum of uninfected animals (n = 6) and Cr infected mice (n = 6). Data are presented as mean values +/− SD. (**d**) The abundance of itaconate, 2-methylsuccinate, glutarate and 2-oxoadipate in supernatant of Cr culture grown in a virulence inducing condition (n = 2). p-values were generated using the two-tailed Mann-Whitney test (**a**-**c**). Source data

Several metabolites showed similar changes in abundance in the Lm and Cr groups, providing clues regarding conserved host metabolic pathways that respond to enteric infection. For instance, the abundance of several secondary bile acids was reduced in both infections (for example, 7-ketodeoxycholate; Fig. 2b,c), suggesting that enteric infection disrupts the production and/or enterohepatic circulation of these microbiota-derived lipids as observed previously^22^. The abundances of metabolites in cluster 3 were elevated in both the Lm and Cr groups (Fig. 1c,d). In total, the amounts of 133 bile metabolites were elevated by these two enteric pathogens relative to the SPF group (Extended Data Fig. 2a and Supplementary Table 4). The common enteric-infection-induced metabolites were enriched in the energy and amino acid categories (Extended Data Fig. 2b,d,e,g,h). Increased abundances of four dicarboxylates, 2-methylsuccinate, glutarate, 2-oxoadipate and itaconate, were particularly prominent in both infections (Fig. 1i–k and Extended Data Fig. 2d,e). Targeted liquid chromatography–tandem mass spectrometry (LC–MS/MS) analysis confirmed the elevation in the abundance of these four dicarboxylates in individual *C. rodentium*-infected animals (Extended Data Fig. 3a). Glutarate and 2-oxoadipate are intermediate products of amino acid metabolism^23^, whereas itaconate is generated from the tricarboxylic acid (TCA) cycle metabolite aconitate^24^. Elevated abundance of itaconate, 2-oxoadipate and glutarate was also observed in the luminal contents of the proximal small intestine (Extended Data Fig. 3b), suggesting that these biliary dicarboxylates can reach the small intestine via transport in bile. However, these dicarboxylates were not elevated in the serum of *C. rodentium*-infected animals (Extended Data Fig. 3c), indicating that the mechanisms controlling the abundances of these compounds in serum and bile may differ and that serum is not the likely source of the dicarboxylates in the proximal intestine. Furthermore, these four dicarboxylates were not detected in supernatants of cultured *C. rodentium* (Extended Data Fig. 3d), suggesting that these compounds were not pathogen derived. Together, these data reveal that the bile metabolome is markedly influenced by the microbiota and altered in shared as well as distinct ways by enteric infections. Below, we investigate potential pathways that generate these biliary compounds and phenotypes linked to itaconate.

### Enteric infections alter the hepatic transcriptome

**Extended Data Fig. 4:**
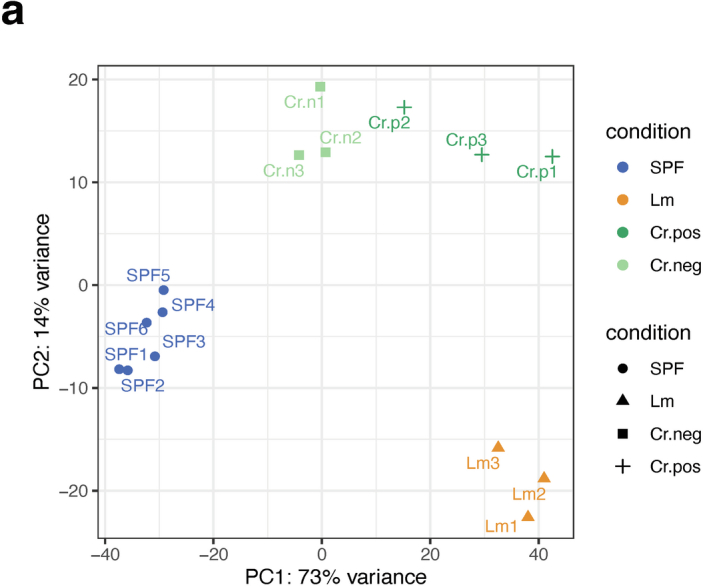
Enteric infections alter hepatic gene expression profiles. (**a**) Principal component analysis (PCA) analysis of liver transcriptomes in uninfected mice (SPF), Lm infected animals at 4 DPI, and Cr infected animals at 10 DPI. *C. rodentium* infected animals were divided into two groups, depending on whether or not they had detectable (Cr positive, Cr.p) or undetectable (Cr negative, Cr.n) *C. rodentium* cfu in the liver.

Given the central role of the liver in bile formation, we profiled the hepatic transcriptome at the same time points as we assayed bile metabolites to investigate whether changes in hepatic gene expression patterns potentially underlie some of the changes observed in the bile metabolome during *L. monocytogenes* and *C. rodentium* infection. Transcriptomes from *C. rodentium*-infected animals were divided into two groups, based on whether the animals had detectable (Cr positive) or undetectable (Cr negative) *C. rodentium* in their livers at the time they were killed, and analysed separately. In total, 3,093 hepatic transcripts had altered abundance in response to *L. monocytogenes* infection (Supplementary Table 5). In *C. rodentium*-infected animals, there were fewer differentially expressed genes (Cr positive: 2,617; Cr negative: 913; Supplementary Table 5). Principal component analysis separated the transcriptional profiles from the four conditions (Extended Data Fig. 4a). Importantly, the transcriptional profiles observed in the Cr-negative group of animals were distinct from those found in SPF mice (Extended Data Fig. 4a), consistent with the idea that enteric infection modulates hepatic transcriptional programmes even in the absence of detectable pathogen dissemination to the liver.

**Fig. 3:**
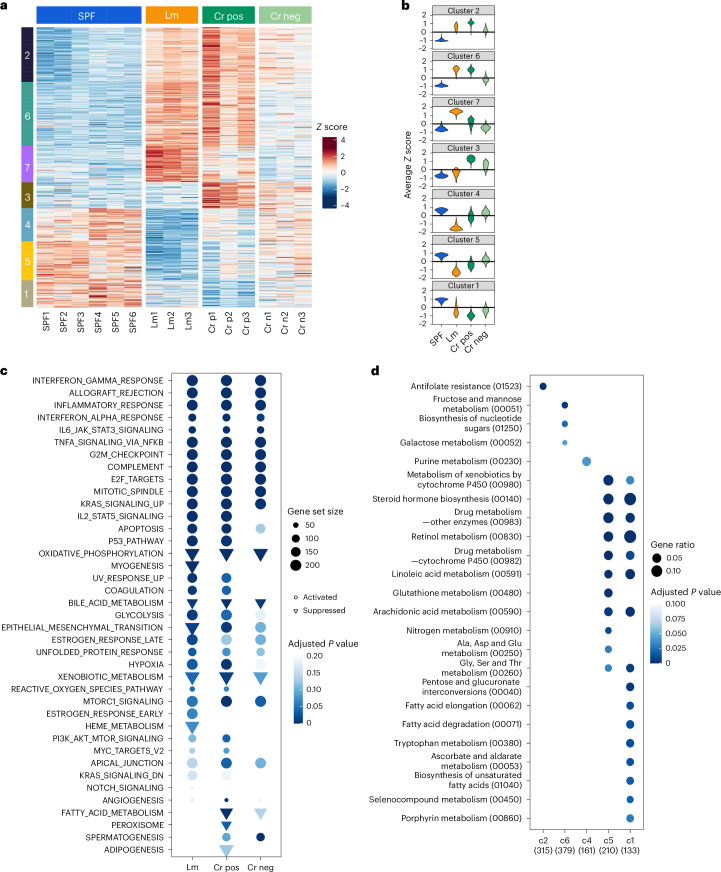
Hepatic transcriptome profiles are altered by enteric infection. **a**, Unsupervised clustering (*k*-medoids) of the differentially expressed genes identified in the four conditions (uninfected (SPF), Lm and *C. rodentium* infected with detectable (Cr pos) or undetectable (Cr neg) CFU in the liver), based on their patterns of relative abundance. Each row represents a gene, and each column represents one mouse. **b**, Violin plot of the distribution of the *Z* scores of the transcript abundance in each cluster. **c**, Gene set enrichment analysis of differentially expressed genes that were identified in Lm versus SPF, Cr liver positive versus SPF and Cr liver negative versus SPF conditions. Gene set size indicates the number of genes associated with specific pathways.The color scale indicates the adjusted *P* value provided by the GSEA function of clusterProfiler (two-tailed Wald test *P* value, adjusted for multiple testing using the procedure of Benjamini and Hochberg). **d**, Functional enrichment analysis of differentially expressed genes using a subset of the KEGG database restricted to metabolism pathways. The categories on the *x*-axis (c2, c6, c5 and c1) correspond to clusters 2, 6, 5, and 1 in **a**. The numbers in parentheses indicate the number of genes associated with each pathway. The color scale indicates the adjusted *P* value provided by the enrichKEGG function of clusterProfiler (two-tailed Wald test *P* value, adjusted for multiple testing using the procedure of Benjamini and Hochberg).

**Fig. 4:**
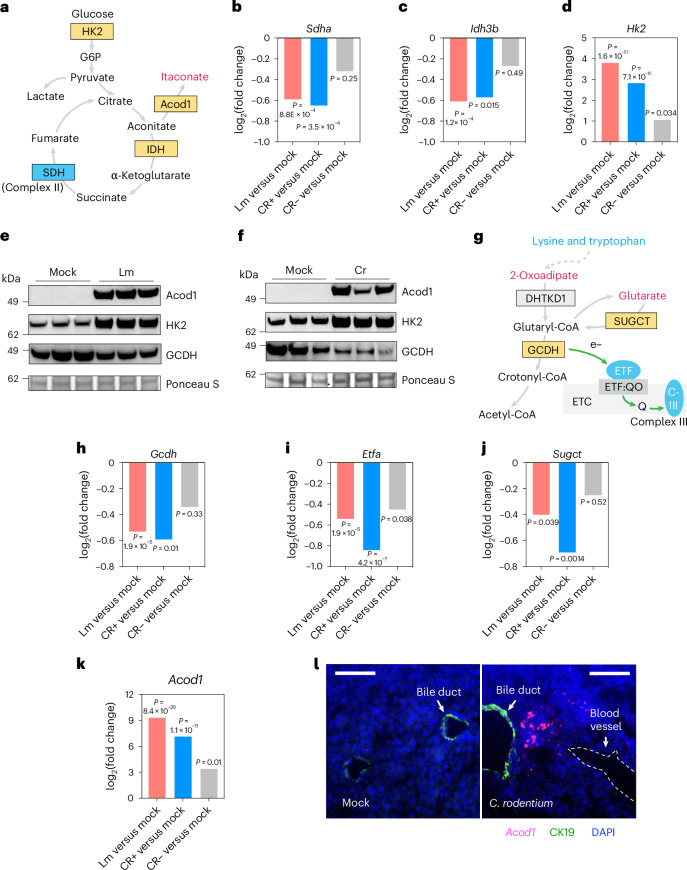
Hepatic gene expression alterations that contribute to infection-associated changes in the abundance of bile dicarboxylates. **a**, Schematic of the pathway for itaconate production. Sugars such as glucose are processed for energy production by HK2 and further converted to pyruvate, which in turn feeds the TCA cycle. Itaconate is produced from the TCA cycle metabolite aconitate by the enzyme Acod1. **b**–**d**, The relative expression levels of TCA cycle enzymes *Sdha* (**b**) and *Idh3b* (**c**), and glycolysis enzyme *Hk2* (**d**) in liver in Lm versus SPF, Cr liver positive versus SPF and Cr liver negative versus SPF conditions. **e**,**f**, The protein level of Acod1, HK2 and GCDH in Lm versus uninfected animals (**e**) and Cr versus uninfected mice (**f**). The signal from Ponceau S-stained samples was used as a loading control. Each experiment was repeated independently twice with similar results. **g**, Proposed model for generation of glutarate and 2-oxoadipate as intermediate products of lysine and tryptophan metabolism. Mutations of GCDH lead to elevations of both glutarate and 2-oxoadipate, whereas mutations of SUGCT lead to elevations of glutarate^23,26,27^. GCDH is a FAD-dependent enzyme that requires the ETF complex to transfer electrons to the ETC; Q, ubiquinone, ETF:QO, electron transfer flavoprotein–ubiquinone oxidoreductase. **h**–**j**, The relative expression level of *Gcdh* (**h**), *Etfa* (**i**) and *Sugct* (**j**) in mouse liver in Lm versus SPF, Cr liver positive versus SPF and Cr liver negative versus SPF conditions. **k**, The relative expression levels of *Acod1* in liver in Lm versus SPF, Cr liver positive versus SPF and Cr liver negative versus SPF conditions. **l**, Representative image of RNA-FISH analysis of *Acod1* transcripts (red) in the liver; CK19 (green), bile duct marker; DAPI (blue), nuclei. The scale bars represent 50 µm. Each experiment was repeated independently twice with similar results. For data in **b**–**d** and **h**–**k**, three animals per condition were used for analysis. Adjusted *P* values were generated using DESeq2 (two-tailed Wald test *P* value, adjusted for multiple testing using the procedure of Benjamini and Hochberg). All genes were statistically differentially expressed in at least two conditions (adjusted *P* < 0.05). Source data

**Extended Data Fig. 5:**
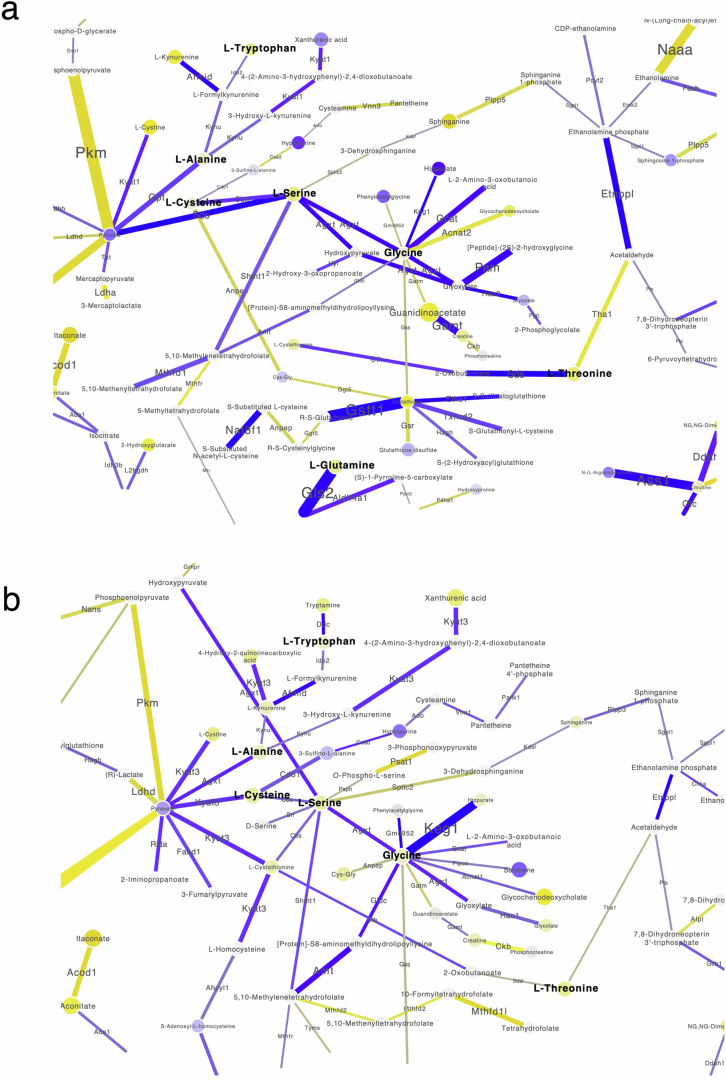
Network analysis of the liver transcriptome and bile metabolome. Integrated analysis of genes and metabolites regulated by *L. monocytogenes* (**a**) and *C. rodentium* (**b**) infection was carried out using ‘Shiny GATOM: integrated analysis of genes and metabolites’ (https://artyomovlab.wustl.edu/shiny/gatom/). The lines represent enzymes and the dots represent metabolites. The size of the dots and the thickness of the lines correlates with statistical significance (bigger or wider represent lower p-values) and the color scale for the dots and lines represents fold change, where red represents increased abundance and green represents decreased abundance.

**Extended Data Fig. 6:**
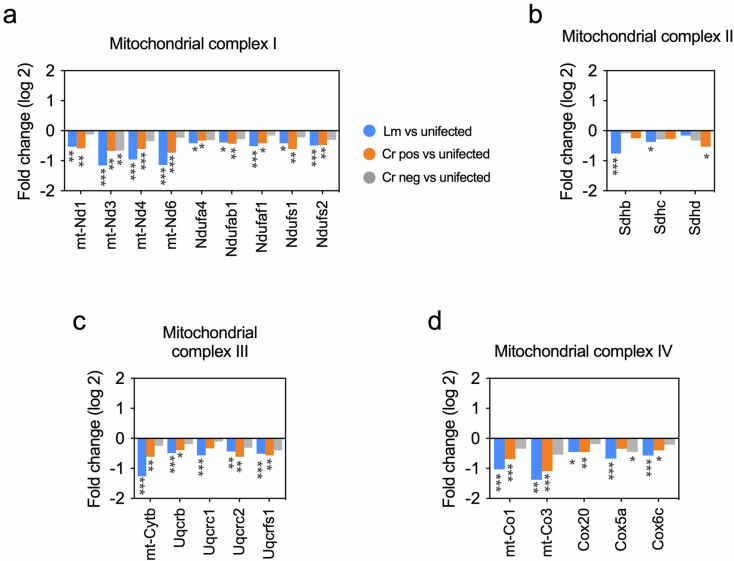
Transcripts of genes encoding liver mitochondrial complexes I, II, III, and IV have reduced abundance during enteric infection. (**a–d**) Differential transcript abundance of liver mitochondrial complex I (**a**), complex II (**b**), complex III (**c**), and complex IV (**d**) in Lm infected, Cr infected liver positive (Cr pos) or liver negative (Cr neg) animals compared to SPF animals. Adjusted p values were generated using DESeq2 (two-tailed Wald test p-value, adjusted for multiple testing using the procedure of Benjamini and Hochberg, * p < 0.05, ** p < 0.01, *** p < 0.001). Source data

Unsupervised clustering was used to partition differentially expressed genes into seven clusters (Fig. 3a,b; adjusted *P* < 0.05). This analysis revealed that there were common changes in hepatic transcriptome profiles associated with infection (Fig. 3a,b). Gene set enrichment analysis and gene-metabolite network analysis also revealed that *L. monocytogenes* and *C. rodentium* infection affected similar biological processes (Fig. 3c and Extended Data Fig. 5). Notably, both enteric infections stimulated hepatic expression of genes linked to inflammation (Fig. 3c), showing that the liver mounts an inflammatory response even in the absence of pathogen dissemination from the intestine. Expression of many genes related to metabolism, including amino acid metabolism (Kyoto Encyclopedia of Genes and Genomes (KEGG) 00260 in Fig. 3d and Extended Data Fig. 5) and detoxification (KEGG 00980 in Fig. 3d), was markedly reduced in both infection models, suggesting that enteric infection depresses a subset of hepatic functional programmes. In particular, infection reduced expression of liver genes involved in oxidative phosphorylation, including all four enzyme complexes in the electron transport chain (ETC) (Extended Data Fig. 6a–d). Similarly, the abundance of transcripts encoding several TCA cycle enzymes (Fig. 4a), including Idh1 and Sdh complex components, was reduced (Fig. 4b,c). By contrast, the abundance of transcripts for several enzymes in the glycolysis pathway was increased (Figs. 3c and 4d); hexokinase (HK2) protein abundance was also elevated, concordant with the transcript data (Fig. 4e,f). Together, these observations suggest that the liver switches from oxidative phosphorylation to glycolysis for energy production during enteric infection.

We next asked whether the hepatic transcriptome could explain the increases we observed in specific previously uncharacterized bile metabolites, in particular the four dicarboxylic acids that were sharply increased in bile samples from infected mice (Fig. 1i–k). Glutarate and 2-oxoadipate are intermediate products of lysine and tryptophan catabolism^23^ (Fig. 4g), but the processes that govern their abundance are not fully characterized. Increased excretion of glutarate and 2-oxoadipate in urine is observed in glutarate aciduria type I (GA-1)^25,26^. GA-1 is caused by loss-of-function mutations in glutaryl-CoA dehydrogenase (GCDH), the enzyme that mediates glutaryl-CoA dissimilation^27^. The abundance of GCDH mRNA and protein was reduced during *L. monocytogenes* and *C. rodentium* infection (Fig. 4e,f,h), probably explaining the corresponding elevated abundance of glutarate and 2-oxodipate in bile. Furthermore, GCDH is a FAD-dependent enzyme and its function relies on the electron transfer flavoprotein (ETF) complex to transfer electrons to complex III in the ETC^28^ (Fig. 4g). *L. monocytogenes* and *C. rodentium* infection decreased the abundance of the mRNAs of the ETF complex (Fig. 4i), as well as transcripts encoding complex III (Extended Data Fig. 6c), potentially further impacting GCDH enzyme activity. The level of glutarate is controlled by succinyl-CoA:glutarate-CoA transferase (SUGCT) that converts glutarate to its CoA form to prevent excretion-mediated carbon loss^29^. Both infections decreased the transcript levels of SUGCT (Fig. 4j), supporting the idea that reduced abundance of GCDH and SUGCT may explain elevations of glutarate in bile of infected animals (Fig. 1i,j). Host pathways for the derivation of 2-methylsuccinate are not clear, but this dicarboxylate may in part be derived from the microbiota^30^.

In contrast to the other dicarboxylates in which reduction of key transcripts appears to account for their induction by infection, *L. monocytogenes* and *C. rodentium* infection substantially stimulated the hepatic expression of *Acod1* (Fig. 4k), the gene encoding the itaconate-synthesizing enzyme aconitate decarboxylase 1 (Acod1, also known as Irg1); concordantly, the abundance of the Acod1 protein was also notably increased in both infection conditions (Fig. 4e,f). Acod1 is mainly expressed in innate immune cells and converts the TCA cycle intermediate aconitate to itaconate (Fig. 4a). RNA fluorescence in situ hybridization (RNA-FISH)-based detection of *Acod1* transcripts in *C. rodentium*-infected animals revealed markedly increased *Acod1* signal in the liver (Fig. 4l). Collectively, these data support the hypothesis that enteric infections shape the bile metabolome by modulating the expression of hepatic metabolic enzymes.

### Itaconate regulates the abundance of intestinal tuft cells

While itaconate has known immunoregulatory activities outside of the intestine^31^, particularly within the intracellular environment where it suppresses inflammasome activation in bone-marrow-derived macrophages^32,33^, the roles of itaconate in the intestine have received little attention. As itaconate abundance was elevated in the bile of both *L. monocytogenes*- and *C. rodentium*-infected animals (Fig. 1i,j and Extended Data Fig. 3a), as well as in the luminal contents of the proximal small intestine of *C. rodentium*-infected animals (Extended Data Fig. 3b), we focused our analyses on unveiling the functions of this dicarboxylate in intestinal homeostasis and defence.

**Fig. 5:**
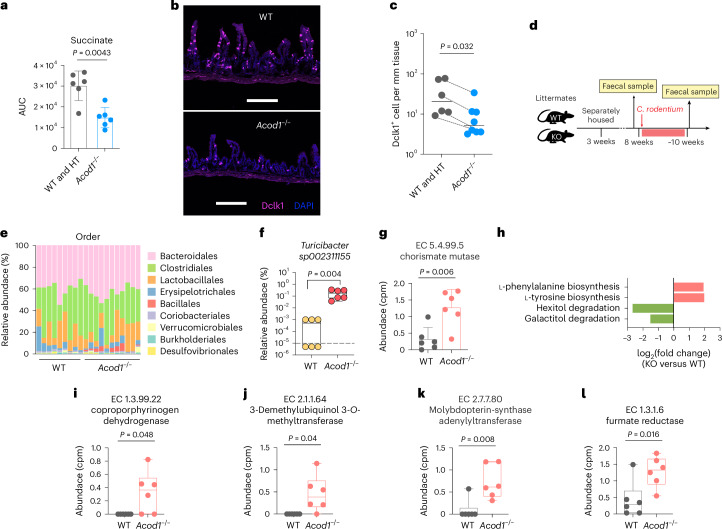
Itaconate regulates tuft cell abundance, and microbiota composition and functional potential. **a**, Abundance of succinate in bile of WT and heterozygote (HT) (*n* = 6) and *Acod1*^−/−^ mice (*n* = 6) after *C. rodentium* infection. AUC, area under the curve. Data are presented as mean ± s.d. **b**, Representative image of the distal ileum of littermates of *Acod1*^+/+^ and *Acod1*^−/−^ mice treated with streptomycin, which elevates intestinal succinate levels^59^. Tuft cells were identified with anti-Dclk1 antibody (pink), and nuclei were stained with DAPI (blue). The scale bars represent 250 µm. **c**, Quantification of tuft cell numbers in the distal ileum. (Data from 4 litters of mice in 3 independent experiments, *n* = 6 for WT and *n* = 8 for *Acod1*^−/−^ mice; lines connect littermates). **d**, Experimental scheme for studying the effects of *Acod1* on faecal microbiota composition. Littermates of mice from *Acod1*^+/−^ heterozygote parents were separately housed according to genotype at 3 weeks of age and challenged with *C. rodentium* at 8 weeks of age. Faecal samples were collected before and after clearance of *C. rodentium* infection (the course of *C. rodentium* infection is ~12–14 days). **e**, Relative abundance of faecal microbiota at the order level after clearance of *C. rodentium* infection (*n* = 9 for WT and *n* = 11 for *Acod1*^−/−^ mice). **f**, Abundance of *Turicibacter sp002311155* in WT (*n* = 6) and *Acod1*^−/−^ mice (*n* = 6) after *C. rodentium* infection identified by shotgun metagenomics (*n* = 6 for WT and *n* = 6 for *Acod1*^−/−^ mice). Whiskers represent minima to maxima. **g**, Relative abundance of chorismate mutase in *Acod1*^−/−^ mice compared with WT mice after *C. rodentium* infection (*n* = 6 for WT and *n* = 6 for *Acod1*^−/−^ mice). cpm, copies per million. Data are presented as mean ± s.d. **h**, Differentially abundant pathways in *Acod1*^−/−^ mice (*n* = 6) compared with those of WT mice (*n* = 6) after *C. rodentium* infection. **i**–**l**, The four most enriched EC families in *Acod1*^−/−^ mice compared with WT mice after *C. rodentium* infection (*n* = 6 for WT and *n* = 6 for *Acod1*^−/−^ mice): EC 1.3.99.22 (**i**), EC 2.1.1.64 (**j**), EC 2.7.7.80 (**k**) and EC 1.3.1.6 (**l**). Data are presented as mean ± s.d. Whiskers represent minima to maxima. *P* values were generated using a two-tailed Mann–Whitney test (**a**, **c**, **f**, **g**, **i**–**I**) or LEfSe, two tailed) (**f**). Source data

**Extended Data Fig. 7:**
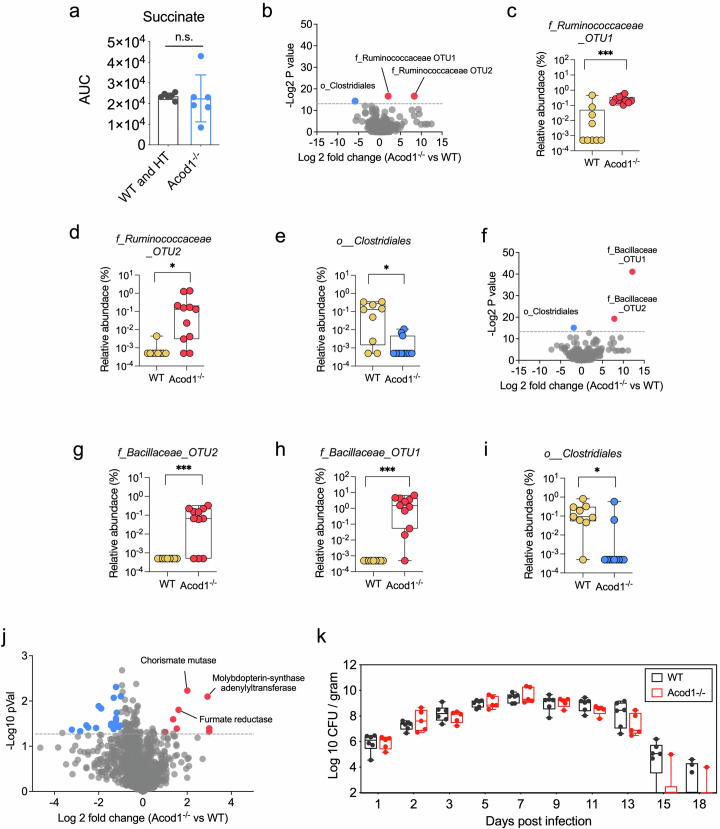
Enteric infection-stimulated bile metabolites regulate gut microbiota composition. (**a**) Abundance of succinate in bile of WT (n = 6) and Acod1^−/−^ mice (n = 6) of uninfected animals. Data are presented as mean values +/− SD. (**b**) Differential abundance of fecal microbiota at the OTU level between WT (n = 9) and Acod1^−/−^ mice (n = 11) before *C. rodentium* infection. (**c–e**) The three most differentially abundant OTUs in Acod1^−/−^ mice (n = 11) compared to WT mice (n = 9) before *C. rodentium* infection. Whiskers represent minima to maxima. (**f**) Differential abundance of fecal microbiota at the OTU level between WT (n = 9) and Acod1^−/−^ mice (n = 11) post *C. rodentium* infection. (**g–i**) Three most differentially abundant OTUs in Acod1^−/−^ mice (n = 11) compared to WT mice (n = 9) post *C. rodentium* infection. Whiskers represent minima to maxima. (**j**) Differential abundance of functions (Enzyme Commissions, ECs) in WT vs Acod1^−/−^ mice post *C. rodentium* infection. (**k**) CFU of *C. rodentium* in the feces of infected WT (n = 6) and Acod1^−/−^ mice (n = 5). p-values were generated by two-tailed Mann-Whitney test (**a**, **j**) or ANCOMBC (two-tailed, **b**-**i**). Source data

One of itaconate’s metabolic effects in immune cells is inhibition of the activity of the tricarboxylic acid cycle enzyme succinate dehydrogenase, leading to accumulation of succinate in culture supernatants^34^. Concordant with these cell-cultured-based observations, we found that the abundance of succinate in the bile of wild-type (WT) mice was markedly higher than in *Acod1*^−/−^ littermates following *C. rodentium* infection (Fig. 5a); however, basal succinate levels were comparable in uninfected WT and *Acod1*^−/−^ animals (Extended Data Fig. 7a). As succinate promotes the proliferation of intestinal tuft cells by signalling through the intestinal epithelial cell surface G-protein coupled receptor Sucnr1 (refs. ^35,36^), we tested whether the itaconate–succinate axis regulates the abundance of these chemosensory cells by comparing the number of tuft cells in *Acod1*^+/+^ and *Acod1*^−/−^ littermates. *Acod1*^+/+^ mice had ~5-fold greater tuft cell abundance than *Acod1*^−/−^ mice in their ilea (Fig. 5b,c). Together, these observations are consistent with a model showing that itaconate stimulation of biliary and/or intestinal succinate levels elevates the abundance of intestinal tuft cells.

### Altered gut microbiota composition in *Acod1*^−/−^ mice

Several bile components, including bile acids, regulate both the abundance and function of intestinal commensal species^37,38^. We investigated whether gut microbiota composition is altered in *Acod1*^−/−^ animals. In these experiments, *Acod1*^+/+^ and *Acod1*^−/−^ littermates were separately housed according to genotype when they were 3 weeks old and the faecal microbiota compositions of the two groups were compared when the animals were ~8 weeks old (Fig. 5d). The 16S rRNA gene profiling showed that several operational taxonomic units (OTUs), including two Ruminococcaceae species and one Clostridiales species, showed marked difference in abundance in the *Acod1*^−/−^ and *Acod1*^+/+^ mice (Extended Data Fig. 7b–e), suggesting that itaconate influences gut microbiota composition at steady state.

As enteric infections disrupt the intestinal microbiota^20^ and we found that the abundance of itaconate in bile increased with enteric infection (Fig. 1i,j), we investigated the composition of the gut microbiota in *Acod1*^−/−^ mice following *C. rodentium* infection. *Acod1*^−/−^ and *Acod1*^+/+^ mice were challenged with *C. rodentium*, and we profiled their faecal microbiota during the recovery phase following pathogen clearance (Fig. 5d). The *Acod1*^−/−^ animals had a marked increase in the abundance of the Bacillales order after *C. rodentium* infection (Fig. 5e) that was mostly due to a >100-fold increase in the abundance of two Bacillaceae species (Extended Data Fig. 7f–h). Shotgun metagenomics enabled identification of one of the species as *Turicibacter sp002311155*, whose abundance was ~300-fold higher in *Acod1*^−/−^ mice (Fig. 5f). By contrast, the abundance of one Clostridiales species was notably lower in *Acod1*^−/−^ mice (Extended Data Fig. 7f,i), suggesting that itaconate helps to maintain its abundance during and/or following perturbations such as *C. rodentium* infection.

We leveraged shotgun metagenomics data to understand how the functional output of the gut microbiome was changed in *Acod1*^−/−^ mice following *C. rodentium* infection. Compared with WT mice, *Acod1*^−/−^ mice had 27 differentially abundant functions (Enzyme Commission (EC)) identified (Extended Data Fig. 7j). Among them, chorismate mutase (EC 5.4.99.5), a key enzyme for both tyrosine and phenylalanine biosynthesis, was enriched in *Acod1*^−/−^ mice (Fig. 5g); pathway abundance analysis also unveiled the elevation of these aromatic amino acid biosynthesis pathways in *Acod1*^−/−^ mice (Fig. 5h). In addition, the abundance of functions that are critical for haem synthesis (Fig. 5i) and microbial anaerobic and aerobic respiration, including enzymes involved in ubiquinone synthesis (Fig. 5j), molybdopterin-synthase activation (Fig. 5k) and fumarate reduction (Fig. 5l), were elevated in *Acod1*^−/−^ mice, suggesting that itaconate inhibits specific functional pathways. Collectively, these data support the idea that itaconate modulates the composition and functional output of the gut microbiome following perturbations such as enteric infection.

### Itaconate promotes host defence against *Vibrio cholerae*

**Fig. 6:**
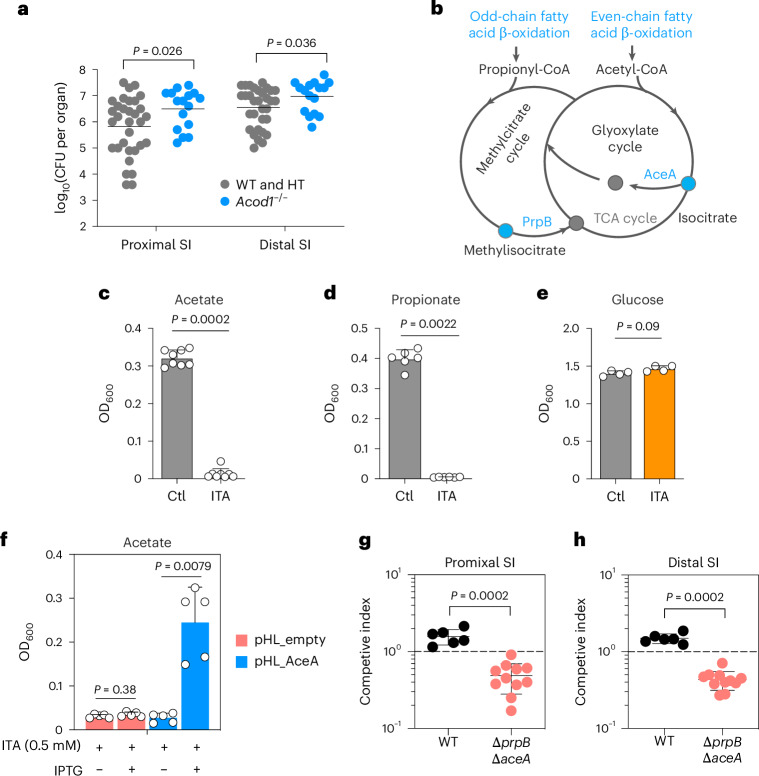
Itaconate confers resistance to *V. cholerae* intestinal colonization. **a**, CFU of *V. cholerae* in the proximal and distal small intestine of infected WT and *Acod1*^−/−^ mice (data from four independent experiments, *n* = 32 for WT and HT, and *n* = 16 for *Acod1*^−/−^ mice). SI, small intestine. Data are presented as mean values. **b**, Scheme for acetate and propionate utilization in *V. cholerae*; AceA, isocitrate lyase; PrpB, methylisocitrate lyase. **c**–**e**, Growth of *V. cholerae* in M9 minimal medium supplemented with 10 mM acetate (**c**), propionate (**d**) or glucose (**e**) as the sole carbon source, in the presence or absence of 10 mM of itaconate (*n* = 8 for acetate, *n* = 6 for propionate and *n* = 4 for glucose). Ctl, control; ITA, itaconate. Data are presented as mean ± s.d. **f**, Growth of *V. cholerae* pHL_empty (vector control) and *V. cholerae* pHL_AceA strain in M9 minimal medium supplemented with 10 mM acetate and 0.5 mM itaconate, in the presence or absence of IPTG (*n* = 5, data represent biological replicates from two independent experiments). **g**,**h**, Competitive index of *V. cholerae* Δ*prpB*Δ*aceA* versus the WT strain in proximal (**g**) and distal (**h**) small intestine of infant mice. CIs were calculated by dividing the ratio of white to blue (Δ*aceA*Δ*prpB*/WT, *n* = 11) colonies in the small intestine by the ratio of white to blue colonies in the inoculum, and compared with the CI of Haiti WT *lacZ*^*−*^ and Haiti WT *lacZ*^*+*^ strain (*n* = 6). *P* values were generated using the two-tailed Mann–Whitney test (**a**, **c**–**h**). Source data

**Extended Data Fig. 8:**
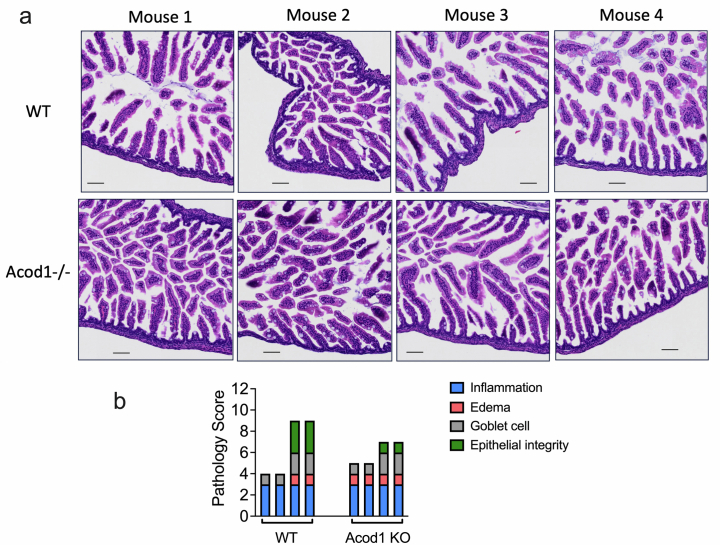
Histological analysis of the intestinal tissues from *Vibrio cholerae* infected WT and Acod1-/- mice. (**a**) Hematoxylin and Eosin (H&E) stained small intestine of WT (top) and Acod1-/- (bottom) mice post *V. cholerae* infection. Scale bar represents 50 µm. Images represent four independent animals in one experiment. (**b**) Combined pathology scores. Each bar represents an individual animal.

Itaconate has antimicrobial activity against several bacterial pathogens, including *Mycobacterium tuberculosis*^24,39^. Because we identified itaconate in the extracellular environment of mouse tissues, including bile and the intestinal lumen (Extended Data Fig. 3a,b), we tested whether this immune metabolite plays a role in host defence against extracellular enteric pathogens. We first challenged *Acod1*^−/−^ and *Acod1*^+/−^ littermates with *C. rodentium* and found no difference in faecal shedding of this pathogen (Extended Data Fig. 7k). As bile metabolites are likely to have higher concentrations in the small intestine than in the colon, we challenged neonatal *Acod1*^−/−^ and *Acod1*^+/−^ littermates with a pathogen that primarily replicates in the small intestine, *V. cholerae*, the agent of cholera^40^. Compared with *Acod1*^+/−^ mice, *Acod1*^−/−^ mice had an ~5 times greater *V. cholerae* burden recovered from their proximal and distal small intestine. (Fig. 6a), suggesting that itaconate restricts *V. cholerae* growth within the small bowel. Histological analysis of intestinal tissues from *V. cholerae*-infected animals suggested that there were no discernable differences between WT and *Acod1*^−/−^ mice (Extended Data Fig. 8), indicating that itaconate-mediated defence does not rely on altering tissue structure.

We hypothesized that itaconate directly inhibits *V. cholerae*’s ability to grow on short-chain fatty acids because studies in *M. tuberculosis* revealed that this dicarboxylate impairs the activity of the pathogen’s isocitrate lyase and methylisocitrate lyase, enzymes that mediate utilization and/or detoxification of short-chain fatty acids (Fig. 6b)^41^. We found that itaconate inhibited *V. cholerae* growth, as it did with *M. tuberculosis* growth, when short-chain fatty acids acetate or propionate were used as the sole carbon source (Fig. 6c,d), but not when glucose was the carbon source (Fig. 6e). Supporting the idea that itaconate inhibition of *V. cholerae* isocitrate lyase (AceA) impairs the pathogen’s growth on acetate, we found that overexpression of AceA restored *V. cholerae* growth in the presence of itaconate (Fig. 6f). These data support the idea that itaconate inhibits the activities of *V. cholerae* AceA and potentially methylisocitrate lyase (PrpB), curbing the glyoxylate and methylisocitrate cycles that govern the utilization of even- and odd-chain fatty acids, respectively (Fig. 6b).

As fatty acid utilization is thought to support robust *V. cholerae* intestinal colonization of infant mice^42^, we tested whether AceA and PrpB contributed to *V. cholerae* growth in the infant mouse intestine. A *V. cholerae* mutant strain (Δ*aceA*Δ*prpB*) lacking both isocitrate lyase and methylisocitrate lyase showed a competitive colonization defect (Fig. 6g,h), supporting the idea that the pathogen relies on fatty acids for robust growth in the host intestine. These data are consistent with the hypothesis that itaconate mediates host defence against *V. cholerae* by impairing the pathogen’s capacity to consume fatty acids in the intestine. Collectively, these observations suggest that itaconate controls the composition of the intestinal epithelium and microbiota as well as intestinal defence.

## Discussion

**Extended Data Fig. 9:**
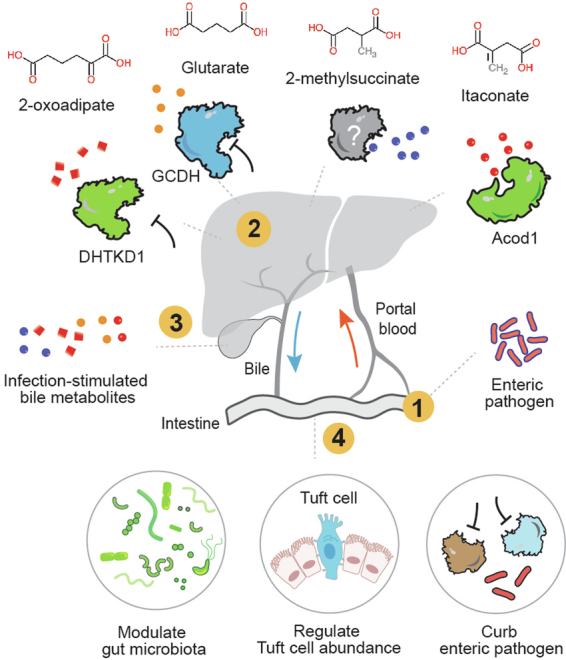
Remodeling of bile composition triggered by infection modifies intestinal components and defense. Proposed model of a gut-liver defense circuit in which intestinal infection stimulates modifications in bile composition that in turn modulate intestinal function. Enteric infection (1) stimulates changes in hepatic transcriptional profiles (2) that alter bile composition (3) that in turn control microbiota and epithelial composition and host defense (4).

Bile has been recognized as a vital body fluid important for the maintenance of health at least since the days of Hippocrates^43^. Here we profiled the mouse bile metabolome using untargeted metabolomics and investigated how the microbiota and enteric infection modulate the bile metabolome. Our findings provide a new perspective on bile function. We discovered that the bile metabolome is composed of hundreds of metabolites and is shaped by the microbiota and remodelled by enteric infection. Moreover, we found that an infection-stimulated bile metabolite modulates the composition of the intestinal epithelium and microbiota as well as intestinal defence. Changes in hepatic transcriptional profiles induced by enteric infection probably explain how infection remodels bile composition. Together, our findings unveil a new interorgan defence circuit embedded in enterohepatic circulation in which intestinal infection stimulates modifications in bile composition that in turn modulate intestinal function (Extended Data Fig. 9).

Our observations suggest that enteric infection leads to the release of signals that enable the intestine to leverage the hepatic capacity to synthesize bile to modulate gut functions including defence (Extended Data Fig. 9). The delivery of infection-stimulated modified bile to the intestine through the common bile duct can be thought of as analogous to the acute phase response, in which infection-stimulated hepatic products are secreted into the blood to aide in systemic defence and tissue repair^4^. However, modification of bile composition in response to enteric infection targets the intestine and represents a previously uncharacterized innate defence circuit to guard the intestine that functions alongside autonomous enteric defence systems^44^. The liver responds to enteric infection even in the absence of detectable pathogen cells replicating in the liver (Fig. 3a and Extended Data Fig. 4a), suggesting that the hepatic sensing mechanisms are at least in part driven by host-derived factors along with microbial-associated products. Identifying the signals and hepatic sensing and regulatory elements that govern the type, magnitude and duration of hepatic-driven changes in bile makeup are key challenges for future studies.

In summary, our findings uncovered the complex and dynamic nature of bile composition and expands knowledge of the homeostatic functions of the liver and bile.

We discovered that bile itaconate abundance increases with infection and that *Acod1*^−/−^ mice have altered microbiota, epithelial composition and defence against an enteric pathogen. However, as *Acod1*^−/−^ mice are not only deficient in bile-derived itaconate, we cannot exclude the possibility that other sources of itaconate could contribute to the intestinal phenotypes we observed. Also, infection-related changes in food intake could influence bile composition, and the dilution effect of bile metabolites in different sections of the intestinal tract is unknown. Finally, we note that the 812 bile metabolites identified here probably represent a subset of the complete bile composition atlas. Many more molecules will undoubtedly be discovered as MS/MS improves^45^.

## Methods

### Animals

All animal experiments were conducted following the protocol (2016N000416) reviewed and approved by the Brigham and Women’s Hospital Institutional Animal Care and Use Committee. SPF C57Bl/6J mice were purchased from the Jackson Laboratory (stock number 000664). The mice were kept in a Harvard Medical School animal facility for at least 72 h before the experiments. GF C57Bl/6J mice were purchased from the Massachusetts Host-Microbiome Center. *Acod1*^−/−^ mice were purchased from the Jackson Laboratory (C57BL/6NJ-Acod1em1(IMPC)J/J, stock number 029340) and bred in the Harvard Medical School animal facility. C57Bl/6 mice with 3 day postnatal infants (P3) were purchased from the Charles River Laboratories (stock number 027) and kept in the Harvard Medical School animal facility until postnatal day 5 (P5). All mice were kept under 12 h light–dark cycles, with lights turned off at 7 p.m. and turned on at 7 a.m. and temperature (68–75 °F) and humidity (50%) controlled. Food and water were given ad libitum.

### Global metabolomic profiling of bile

Female SPF mice (9–10 weeks old) were orally inoculated with *L. monocytogenes* or *C. rodentium* following previously described protocols^17,46^. Briefly, mice were deprived of food for 6 h, lightly sedated with isoflurane inhalation and oro-gastrically inoculated with 3 × 10^9^ CFU of *L. monocytogenes* 10403S InlA^m^ strain in a 300 µl mixture of 200 mM CaCO_3_ in PBS or with 1 × 10^9^ CFU *C. rodentium* ICC168 strain in 200 µl PBS using 18 G flexible feeding needles (DT 9928, Braintree Scientific). Uninfected SPF mice were inoculated with 200 µl of PBS. Animals were killed 4 days after *L. monocytogenes* infection and 10 days after *C. rodentium* infection. Bile samples were collected from animals at approximately 3 p.m.; at this time of the day, the bile volume of uninfected mice showed some variation but was not empty. Both *L. monocytogenes* and *C. rodentium* infection increased bile volume. Bile samples were collected from the gall bladder using insulin syringes (BEC-309311, Becton Dickinson), filtered with 0.22 µm centrifuge tube filters (8160, Corning), snap-frozen in liquid nitrogen and stored in −80 °C until analysis. To obtain a minimal volume of 60 µl of bile for global metabolomic profiling, samples were generated by pooling bile from 3 to 5 SPF mice, 3 or 4 *L. monocytogenes*-infected or *C. rodentium*-infected mice, and 2 or 3 GF mice. The liver and bile of *L. monocytogenes*-infected mice and the liver and colon of *C. rodentium*-infected mice were used for bacterial burden enumeration.

Bile samples were processed and analysed by Metabolon for global metabolomic profiling. Briefly, to remove protein and recover chemically diverse metabolites, the samples were combined with methanol and vigorously mixed for 2 min (Glen Mills GenoGrinder 2000) followed by centrifugation. The extract was divided equally into four fractions for analysis. Detailed parameters for chromatography and mass spectrometry analysis were carried out as described previously^47^ and provided in Supplementary Table 1f. All the studies were conducted using the following platform: Waters ACQUITY ultra-performance liquid chromatography and Thermo Scientific Q-Exactive high-resolution and accurate mass spectrometry interfaced with a heated electrospray ionization (HESI-II) source and Orbitrap mass analyser operated at 35,000 mass resolution. Briefly, the extract was divided equally into four fractions for metabolite identification using four separate platforms (Supplementary Table 1f). To ensure injection and chromatographic consistency, a series of internal standards at fixed concentrations were added to the reconstitution solvent (Supplementary Table 1f). The first aliquot of sample was analysed using acidic positive ion conditions, a method chromatographically optimized for more hydrophilic compounds. Water and methanol, containing 0.05% perfluoropentanoic acid and 0.1% formic acid, were used to elute the extract from a C18 column (Waters UPLC BEH C18—2.1 × 100 mm, 1.7 µm). Acidic positive ion conditions were also used for analysing the second aliquot. In this method, the extract was gradient eluted from the same C18 column mentioned before using methanol, acetonitrile, water, 0.05% perfluoropentanoic acid and 0.01% formic acid and was operated at an overall higher organic content. A third aliquot was analysed using a separate dedicated C18 column with 6.5 mM ammonium bicarbonate at pH 8 using basic negative ion optimized conditions. The fourth aliquot of sample was analysed using a negative ionization condition and was eluted from a HILIC column (Waters UPLC BEH Amide 2.1 × 150 mm, 1.7 µm) using a gradient consisting of water and acetonitrile with 10 mM ammonium formate at pH 10.8. The MS analysis alternated between MS and data-dependent MS scans using dynamic exclusion, and the scan range varied from 70 to 1,000 m/z (Supplementary Table 1f).

After the raw data were extracted, the compounds were identified by comparison with library entries of purified standards. Metabolon uses a reference library based on authenticated standards, which contains key information including mass-to-charge ratio (m/z*)*, retention time index and chromatographic data (including MS/MS spectral data) on all molecules present in the library. Metabolon’s Laboratory Information Management System maintains more than 3,300 commercially available purified standard compounds, and these compounds were used for analysis on all platforms for determination of their analytical characteristics. Biochemical identifications are based on three criteria: retention index within a narrow retention time index window of the proposed identification, accurate mass match to the library ±10 ppm and the MS/MS forward and reverse scores between the experimental spectrum and authentic standards (ions present in the library spectrum). Biochemicals were distinguished and differentiated by the collective information of all three data points. Area under the curve was used to quantify the peaks. The peaks were quantified using area under the curve and reported as ‘original scale’ data, and significance was determined using a Wilcoxon rank test. For metabolite clustering, ‘original scale’ data were used for calculating *Z* scores. The clustering of the metabolites was performed using the partitioning around medoids (pam) function in the R package cluster (v2.1.4) with a parameter ‘*k* = 7’.

### Metabolite pathway enrichment analyses

We used the Metabolon in-house pathway database to identify enriched pathways representing differentially abundant metabolites. The enrichment score is calculated using the following formula:

$${\text{Enrichment value}}=\frac{\frac{k}{m}}{\frac{n-k}{N-m}}$$

where *m* = number of metabolites in the pathway, *k* = number of significant metabolites in the pathway, *n* = total number of significant metabolites and *N* = total number of metabolites. We arbitrarily set *k* > 4 to eliminate small pathways, which can confound these analyses. The enrichment scores for all the pathways are shown in Supplementary Table 3.

### Quantification of dicarboxylates in bile, serum and proximal intestinal contents

For quantification of the abundance of the dicarboxylates itaconate, 2-methylsuccinate, glutarate and 2-oxoadipate in different anatomical sites, female SPF mice (9–10 weeks old) were oro-gastrically inoculated with *C. rodentium* or PBS as described above. Bile, proximal intestinal contents and blood samples were collected from individual infected mice at 10 dpi. Bile samples were processed as described above; blood samples were kept at room temperature for 1 h and centrifuged at 12,000 *g* for 5 min to obtain serum. Samples were prepared for LC–MS/MS analysis following the sample preparation guideline in ref. ^48^. Briefly, one volume of bile or serum was combined with four volumes of cold methanol (−80 °C), gently mixed and incubated at −80 °C for 6 h. Metabolites in the proximal small intestine contents were extracted using 80% methanol; small pestles were used to grind the contents. Samples were vortexed and incubated at −80 °C for 4 h. Samples were then centrifuged at 14,000 *g* for 10 min (4 °C), and supernatants were transferred to new tubes and lyophilized to pellets without heat. Samples were analysed in the metabolomics core facility at the Beth Israel Deaconess Medical Center using a 6500 QTRAP LC–MS/MS system using the selected reaction monitoring (SRM) mode^48^. Commercially available glutarate (G3407, Sigma), itaconate (I29204, Sigma), 2-methylsuccinate (AAH6096714, Fisher) and 2-oxoadipate (75447, Sigma) were used as chemical standards. SRM data for each metabolite were acquired in either negative or positive ionization modes, and the Q1–Q3 transition for each metabolite was selected (itaconate (Q1 129.1, Q3 85.1), 2-oxoadipate (Q1 159.0, Q3 59.2), 2-methylsuccinate (Q1 131.0, Q3 87.0), glutarate (Q1 131.0, Q3 41.0)). The Q3 peak areas in the SRM data were integrated using MultiQuant 3.0 for quantification across the sample set. Serial diluted chemical standards of itaconate and 2-methylsuccinate were used to generate standard curves to infer the absolute concentration of these two compounds.

### Quantification of dicarboxylates in supernatants from *C. rodentium* culture

A 1 ml overnight culture of *C. rodentium* was washed twice with DMEM with glucose, glutamine and HEPES (Gibco, 12430); inoculated into 50 ml of DMEM; and incubated in an incubator at 37 °C with 5% CO_2_ and without shaking for 4 h, a growth condition that has been shown to induce expression of the pathogen virulence programme^49^. Subsequently, 1 ml of bacteria was collected and centrifuged at 8,000 *g* for 2 min; the supernatants were transferred to new tubes and centrifuged at 12,000 *g* for 2 min. The supernatants were used for LC–MS/MS analysis.

### Hepatic RNA sequencing

C57BL/6J mice were infected with *L. monocytogenes* or *C. rodentium* following the protocols described above. RNAs were extracted from the liver samples using Trizol (Thermo Fisher), and RNA-sequencing (RNA-seq) libraries were prepared using the KAPA RNA Hyperprep Kit (Roche). The libraries were sequenced on an Illumina NextSeq 550 instrument with paired-end runs of 2 × 75 bp, and FASTQ files were generated by Illumina’s FASTQ generation pipeline (V1.0.0). Low-quality bases and the adaptors were trimmed using Trim Galore (v0.6.6) with a paired option. The reads were mapped to the mouse reference genome (mm10) using STAR v2.7.3a with default parameters, and the gene-level raw read count matrix was obtained using the featureCounts function in the subread (v2.0.0). Differentially expressed genes were identified using the R-package DESeq2 (v1.36.0) with an adjusted *P* < 0.05 and an absolute fold change of 1, and the clustering was performed using the pam function in the R-package cluster (v2.1.4) with a parameter ‘*k* = 7’. Pathway analysis was performed by the R-package clusterProfiler (v4.4.4) with gene sets from msigdbr R-package (v7.5.1) or KEGG metabolism pathways.

### Metabolic network analysis

Metabolic network analysis (integrated analysis of genes and metabolites) was performed using Shiny GATOM (https://artyomovlab.wustl.edu/shiny/gatom/) with the following parameters: KEGG network, network type; atoms, network topology; *P* value threshold, scoring parameter for genes; *P* value threshold, scoring parameter for metabolites with the thresholds set to −4. The fold changes in input data for genes were shrunk using the function lfcShrink (type is ‘apeglm’) in the R-package DESeq2. The fold changes for metabolites were ‘Fold of Change’ of *L. monocytogenes*-infected or *C. rodentium*-infected mice compared with SPF mice reported by Metabolon.

### Quantification of succinate in bile

Littermates of female mice (10–14 weeks old) from *Acod1*^+/−^ parents were either infected with *C. rodentium* following the protocol described above or maintained uninfected in a separate cage. Bile samples were collected from uninfected animals (basal level) and infected mice at 10 dpi, and prepared following the protocol described above (quantification of dicarboxylates). The abundance of succinate in samples was quantified using a polar metabolite detection pipeline on a 6500 QTRAP LC–MS/MS system at the mass spectrometry core facility of Beth Israel Deaconess Medical Center.

### Tuft cell immunohistology

Littermates of *Acod1*^−/−^, *Acod1*^+/−^ and *Acod1*^+/+^ adult male mice (10–16 weeks old) were used for these experiments. Mice were administered 200 µl of streptomycin (20 mg) in water daily via oral gavage using 18 G flexible feeding needles (DT 9928, Braintree Scientific) for 5 days and euthanized at day 7 (ref. ^36^). Sections of the distal ileum 1.5 cm long were dissected, fixed with 4% paraformaldehyde for 2 h at room temperature and transferred to 30% sucrose in PBS at 4 °C overnight. The samples were embedded in 1:2.5 of 30% sucrose and optimal cutting temperature compound solution and cut into 10 µm sections. The slides were washed three times with PBS and blocked with 5% normal goat serum and 0.3% Triton X-100 for 1 h at room temperature. The slides were incubated with rabbit anti-Doublecortin-like and CAM kinase-like 1 (DCAMK) polyclonal antibody (1:500 dilution, ab31704, Abcam) at 4 °C overnight and Alexa-594 goat anti-rabbit IgG secondary antibody (1:1,000 dilution, A-11072, Thermo Fisher) for 1 h at room temperature. DNA was labelled with DAPI (P36935, Thermo Fisher). Images were captured using a Leica Stellaris Confocal Microscope at the Microscopy Resources on the North Quad (MicRoN) core of Harvard Medical School. For quantification of tuft cell frequency, the number of Dclk1-positive cells was enumerated in a 2.5 mm-long representative tissue and presented as number of tuft cells per mm tissue.

### Analysis of faecal microbiota composition

Littermates of female mice from *Acod1*^+/−^ parents were separately housed according to their genotypes (*Acod1*^−/−^ versus *Acod1*^+/−^ and *Acod1*^+/+^) at 3 weeks of age and infected with *C. rodentium* at 8 weeks of age following the protocol described above. Faecal pellets were collected 1 day before *C. rodentium* infection and after pathogen clearance (~14 dpi). Genomic DNA was extracted from faecal samples following the method described previously^50^. The V3–V4 region of 16S rRNA was amplified using primers 341F and 805R (Supplementary Table 4), and libraries were prepared using the Nextera XT Index Kit v2 (Illumina). The libraries were sequenced on an Illumina Mi-Seq instrument using Miseq Reagent Kit v3 (600 cycles) with paired-end runs. The Qiime2 pipeline^51^ was used to process the reads, and the Greengene reference library was used for taxonomy mapping. Differential abundance of OTUs was analysed using R package ANCOMBC^52^.

For shotgun metagenomic sequencing, DNA samples were prepared as described above and sent for sequencing in SeqCenter using 2 × 150-cycle paired-end runs. Data were processed for quality control using KneadData^53^. For taxonomical profiling, reads were processed using a *k*-mer method in Kraken2 (ref. ^54^) using a Kraken2 database from the Mouse Gastrointestinal Bacterial Catalogue project^55^. Taxonomical abundance was estimated using Bracken^56^. Microbial functional profiling was performed using Humann3 (ref. ^53^).

### Itaconate inhibition of *V. cholerae* growth in culture

Growth of *V. cholerae* in defined nutrient conditions was analysed using M9 minimal medium containing 1 mM MgSO_4_, 0.3 mM CaCl_2_ and one carbon source as indicated: acetate (10 mM) or glucose (0.5%). When propionate (10 mM) was used as a carbon source, 1× trace element solution (FeCl_2_ (5 µm), MnSO_4_ (50 µm), ZnSO_4_ (1 µm), CuSO_4_ (0.1 µm), CoCl_2_ (0.1 µm), H_3_BO_3_ (0.1 µm), Na_2_MoO_4_ (0.1 µm)) was added. To test the growth inhibitory effect of itaconate, culture mediums were supplemented with a final concentration of 10 mM itaconate and the pH was adjusted to 7 using NaOH. *V. cholerae* were grown in LB broth to OD_60_ ~1.2; 1 ml of *V. cholerae* culture was pelleted by centrifugation at 8,000 *g*, washed twice with M9 minimal medium and resuspended in 1 ml of M9 minimal medium, and 5 µl was inoculated into 1 ml medium of the indicated conditions. All cultures were grown at 37 °C with shaking. OD_600_ was measured at 24 h (for growth in acetate or glucose) or 64 h (for growth in propionate) after inoculation. For the overexpression experiment, the *V. cholerae* pHL_empty strain and *the V. cholerae* pHL_AceA strain were prepared as described above and inoculated into M9 minimal medium supplemented with 10 mM acetate and 0.5 mM itaconate, in the presence or absence of 0.3 mM IPTG. OD_600_ was measured 54 h after inoculation.

### Infection of *Acod1*^−/−^ mice with *V. cholerae*

Littermates of infant mice at postnatal day 5 (P5) from *Acod1*^+/−^ breeding parents were orally inoculated with Haiti WT *V. cholerae*^57^ (total 10^5^ CFUs) in 50 µl LB. Pups were euthanized at 20 h postinfection. The proximal and distal regions of the small intestine were dissected, homogenized and plated on LB streptomycin (Sm) plates for CFU enumeration. Tail samples were used for genotyping.

### Construction of *V. choleraeΔaceAΔprpB*

The *V. cholerae* Haiti Δ*aceA*Δ*prpB* strain was created using allelic exchange as previously described^57^. Briefly, the Haiti WT or Haiti Δ*aceA V. cholerae* strain was conjugated with MFDpir *E. coli* harbouring the suicide plasmid pCVD442 that carried the upstream and downstream genomic region (~700 bp each; Supplementary Table 4) of the targeted gene. The donor and recipient strain were mixed at a 1:1 ratio and incubated at 37 °C for 4 h. To obtain the single crossovers, conjugation reactions were streaked on LB plates with Sm (200 µg ml^−1^) and carbenicillin (Cb; 50 µg ml^−1^). Double crossovers were isolated by restreaking single cross-over colonies on LB plates with 10% sucrose and incubating at room temperature for 2 days. Duplicate patching was used to examine the Cb resistance of the colonies, and the correct double cross-over was identified by screening Sm-resistant and Cb-sensitive colonies using colony PCR.

### *V. cholerae* intestinal competition assay

Overnight cultures of *V. cholerae* Haiti WT *lacZ*^*+*^ and Δ*aceA*Δ*prpB lacZ*^−^ strain were washed once with PBS and diluted 1:1,000 in PBS. Pups at postnatal day 5 (P5) were orally inoculated with a 1:1 mixture (total 10^5^ CFUs) of WT and the Δ*aceA*Δ*prpB V. cholerae* strain in 50 µl PBS. Animals were euthanized at 20 h postinfection. Proximal and distal sections of the small intestine were dissected, homogenized and plated on LB + Sm/X-gal for blue–white colony counting. Competition indices (CIs) were calculated by dividing the ratio of white to blue (Δ*aceA*Δ*prpB* /WT) colonies in the small intestine by the ratio of white to blue colonies in the inoculum, and compared with the CIs of the Haiti WT *lacZ*^−^ and Haiti WT *lacZ*^*+*^ strains.

### RNAscope detection of *Acod1*

Liver samples from *C. rodentium*-infected or PBS-treated animals were embedded in 1:2.5 of 30% sucrose and optimal cutting temperature compound solution, cut into 10 µm sections and stored at −80 °C. Staining followed the fresh frozen tissue protocol provided by ACDbio. Briefly, the slides were fixed with 4% paraformaldehyde that was pre-cooled to 4 °C and incubated at 4 °C for 10 min and washed three times with PBS. The tissue was digested with protease IV and hybridized with an Acod1 probe (stock 450241, ACDbio) and then stained with Opal 570. Slides were incubated with rabbit anti-CK19 monclonal antibody (1:500 dilution, ab52625, Abcam) at 4 °C overnight and Alexa-488 goat anti-rabbit IgG secondary antibody (1:1,000 dilution, A-11072, Thermo Fisher) for 1 h at room temperature. DNA was labelled with DAPI (P36935, Thermo Fisher). Images were captured using a Leica Stellaris Confocal Microscope at the MicRoN core of Harvard Medical School.

### Western blot

Liver samples from *C. rodentium*-infected, *L. monocytogenes*-infected and uninfected animals were homogenized in RIPA buffer (Thermo Fisher, 89900) with Complete Mini Protease Inhibitor (Roche, 11836170001) and centrifuged at 12,000 *g* for 5 min; supernatants were used for western blot analysis. Proteins were separated using NuPAGE 4–12% Bis-Tris Protein Gels (Thermo Fisher, NP0323BOX) and then transferred to nitrocellulose membranes (Thermo Fisher, IB23002). Western blot analysis was carried out using rabbit anti-Acod1 polyclonal antibody (1:1,000 dilution, 17805S, Cell Signaling), rabbit anti-HK2 polyclonal antibody (1:5,000 dilution, Proteintech, 22029-1-AP), rabbit anti-GCDH polyclonal antibody (1:1,000 dilution, AV43559, Sigma) and peroxidase-conjugated goat anti-rabbit secondary antibody (1:5,000 dilution, A4914, Sigma). Chemiluminescent signals were developed with West Pico PLUS Chemiluminescent Substrate (34580, Thermo Fisher) and imaged in the ChemiDoc Touch system (Bio-Rad).

### Statistical analysis

Details of the statistical methods used to analyse each experiment are presented in the figure legends. The ACOMBC, DESeq2 package, GSEA function and enrichKEGG function of clusterProfiler were used, and the Wilcoxon rank test was performed, in R; Mann–Whitney tests were performed in Prism; the Linear Discriminant Analysis Effect Size (LEfSe) was performed in the Conda environment. *P* values generated by Mann–Whitney tests were not corrected for multiple testing.

The sample sizes for the experiments were not predetermined using statistical methods. For untargeted metabolomic analysis, the samples we used were similar to those of a previous study^58^. For metabolomic data analysis, data normality was formally tested, leading to the use of a nonparametric analysis method. For infection experiments, animals were randomly assigned into groups. Animal genotype blinding was used for the *V. cholerae* intestinal colonization assay (*Acod1*^−/−^ and WT animals). Histology analyses of slides stained with haematoxylin and eosin were performed by a pathologist blinded to the experimental scheme. Sample collection and processing for other experiments were not performed in a blinded fashion. No animals were excluded from the analysis.

### Reporting summary

Further information on research design is available in the Nature Portfolio Reporting Summary linked to this article.

## Supplementary information

Reporting Summary

Supplementary Tables 1–6 A descriptive caption is given on top of each table.

## Source data

Source Data Fig. 1 Statistical source data for Fig. 1i,j.

Source Data Fig. 2 Statistical source data for Fig. 2a–c.

Source Data Fig. 4 Unprocessed western blots and/or gels.

Source Data Fig. 5 Statistical source data.

Source Data Fig. 6 Statistical source data.

Source Data Extended Data Fig. 1 Statistical source data.

Source Data Extended Data Fig. 2 Statistical source data.

Source Data Extended Data Fig. 3 Statistical source data.

Source Data Extended Data Fig. 6 Statistical source data.

Source Data Extended Data Fig. 7 Statistical source data.

## Data Availability

Raw sequencing reads for liver transcriptome analysis are available at Gene Expression Omnibus (GEO accession number GSE227180). Raw sequencing reads for 16S and shotgun metagenomic analysis are available at the Sequencing Read Archive (SRA accession number PRJNA947233). Raw metabolomic data from Metabolon are provided in Supplementary Table 1a. Source data are provided with this paper.

## Code availability

The code for this paper is available via GitHub at https://github.com/yh766/NMICROBIOL_Bile_2024.

## References

[1] Diener, C. et al. Genome–microbiome interplay provides insight into the determinants of the human blood metabolome. *Nat. Metab.* **4**, 1560–1572 (2022). 36357685 10.1038/s42255-022-00670-1 PMC9691620

[2] Quinn, R. A. et al. Global chemical effects of the microbiome include new bile–acid conjugations. *Nature* **579**, 123–129 (2020). 32103176 10.1038/s41586-020-2047-9 PMC7252668

[3] Dekkers, K. F. et al. An online atlas of human plasma metabolite signatures of gut microbiome composition. *Nat. Commun.* **13**, 5370 (2022). 36151114 10.1038/s41467-022-33050-0 PMC9508139

[4] Ebersole, J. L. & Cappelli, D. Acute-phase reactants in infections and inflammatory diseases: acute-phase reactants in infections and inflammatory diseases. *Periodontol. 2000* **23**, 19–49 (2000). 11276764 10.1034/j.1600-0757.2000.2230103.x

[5] Mantovani, A. & Garlanda, C. Humoral innate immunity and acute-phase proteins. *N. Engl. J. Med.* **388**, 439–452 (2023). 36724330 10.1056/NEJMra2206346 PMC9912245

[6] Boyer, J. L. Bile formation and secretion. *Compr. Physiol.*10.1002/cphy.c120027 (2013). 10.1002/cphy.c120027 PMC4091928 23897680

[7] Boyer, J. L. in *Physiology of Membrane Disorders* 2nd edn (eds Andreoli, T. E. et al.) Ch. 35 609–636 (Springer, 1986).

[8] Funabashi, M. et al. A metabolic pathway for bile acid dehydroxylation by the gut microbiome. *Nature* **582**, 566–570 (2020). 32555455 10.1038/s41586-020-2396-4 PMC7319900

[9] McKenney, P. T. et al. Intestinal bile acids induce a morphotype switch in vancomycin-resistant *Enterococcus* that facilitates intestinal colonization. *Cell Host Microbe* **25**, 695–705.e5 (2019). 31031170 10.1016/j.chom.2019.03.008 PMC6939634

[10] Stacy, A. et al. Infection trains the host for microbiota-enhanced resistance to pathogens. *Cell* **184**, 615–627.e17 (2021). 33453153 10.1016/j.cell.2020.12.011 PMC8786454

[11] Wikoff, W. R. et al. Metabolomics analysis reveals large effects of gut microflora on mammalian blood metabolites. *Proc. Natl Acad. Sci. USA* **106**, 3698–3703 (2009). 19234110 10.1073/pnas.0812874106 PMC2656143

[12] Moreau, F. et al. 1178-P: portal vein metabolites as intermediate regulators of the gut microbiome in insulin resistance. *Diabetes*10.2337/db21-1178-p (2021). 10.1016/j.cmet.2025.08.005 PMC12529558 40914155

[13] Suzuki, R. Influence of intestinal microorganisms on the metabolism of bile acids in mice. *Keio J. Med.* **19**, 73–86 (1970). 5482419 10.2302/kjm.19.73

[14] Eyssen, H. J., Parmentier, G. G. & Mertens, J. A. Sulfated bile acids in germ‐free and conventional mice. *Eur. J. Biochem.* **66**, 507–514 (1976). 954753 10.1111/j.1432-1033.1976.tb10576.x

[15] Selwyn, F. & Klaassen, C. D. Characterization of bile acid homeostasis in germ‐free mice. *FASEB J.* **26**, 1155.1 (2012).

[16] Hu, H. et al. Gut microbiota promotes cholesterol gallstone formation by modulating bile acid composition and biliary cholesterol secretion. *Nat. Commun.* **13**, 252 (2022). 35017486 10.1038/s41467-021-27758-8 PMC8752841

[17] Zhang, T. et al. Deciphering the landscape of host barriers to *Listeria monocytogenes* infection. *Proc. Natl Acad. Sci. USA* **114**, 6334–6339 (2017). 28559314 10.1073/pnas.1702077114 PMC5474794

[18] Louie, A., Zhang, T., Becattini, S., Waldor, M. K. & Portnoy, D. A. A multiorgan trafficking circuit provides purifying selection of *Listeria monocytogenes* virulence genes. *mBio* **10**, e02948-19 (2019). 31848289 10.1128/mBio.02948-19 PMC6918090

[19] Camilli, A., Tilney, L. G. & Portnoy, D. A. Dual roles of plcA in *Listeria monocytogenes* pathogenesis. *Mol. Microbiol.* **8**, 143–157 (1993). 8388529 10.1111/j.1365-2958.1993.tb01211.x PMC4836944

[20] Hopkins, E. G. D., Roumeliotis, T. I., Mullineaux-Sanders, C., Choudhary, J. S. & Frankel, G. Intestinal epithelial cells and the microbiome undergo swift reprogramming at the inception of colonic *Citrobacter rodentium* infection. *mBio* **10**, e00062-19 (2019). 30940698 10.1128/mBio.00062-19 PMC6445932

[21] Ichikawa, S., Sakiyama, H., Suzuki, G., Hidari, K. I. & Hirabayashi, Y. Expression cloning of a cDNA for human ceramide glucosyltransferase that catalyzes the first glycosylation step of glycosphingolipid synthesis. *Proc. Natl Acad. Sci. USA* **93**, 12654 (1996). 8901638 10.1073/pnas.93.22.12654 PMC38048

[22] Gautam, A. et al. Metabolomic analyses reveal lipid abnormalities and hepatic dysfunction in non-human primate model for *Yersinia pestis*. *Metabolomics* **15**, 2 (2018). 30830480 10.1007/s11306-018-1457-2 PMC6311182

[23] Niska-Blakie, J. et al. Knockout of the non-essential gene SUGCT creates diet-linked, age-related microbiome disbalance with a diabetes-like metabolic syndrome phenotype. *Cell. Mol. Life Sci.* **18**, e1800093 (2019). 10.1007/s00018-019-03359-z PMC7426296 31722069

[24] Michelucci, A. et al. Immune-responsive gene 1 protein links metabolism to immunity by catalyzing itaconic acid production. *Proc. Natl Acad. Sci. USA* **110**, 7820–7825 (2013). 23610393 10.1073/pnas.1218599110 PMC3651434

[25] Bijarnia, S. et al. Glutaric aciduria type I: outcome following detection by newborn screening. *J. Inherit. Metab. Dis.* **31**, 503–507 (2008). 18683078 10.1007/s10545-008-0912-z

[26] Leandro, J. et al. Deletion of 2-aminoadipic semialdehyde synthase limits metabolite accumulation in cell and mouse models for glutaric aciduria type 1. *J. Inherit. Metab. Dis.* **43**, 1154–1164 (2020). 32567100 10.1002/jimd.12276

[27] Christensen, E. Improved assay of glutaryl-CoA dehydrogenase in cultured cells and liver: application to glutaric aciduria type I. *Clin. Chim. Acta* **129**, 91–97 (1983). 6687844 10.1016/0009-8981(83)90155-9

[28] Henriques, B. J., Olsen, R. K. J., Gomes, C. M. & Bross, P. Electron transfer flavoprotein and its role in mitochondrial energy metabolism in health and disease. *Gene* **776**, 145407 (2021). 33450351 10.1016/j.gene.2021.145407 PMC7949704

[29] Marlaire, S., Schaftingen, E. V. & Veiga-da-Cunha, M. C7orf10 encodes succinate-hydroxymethylglutarate CoA-transferase, the enzyme that converts glutarate to glutaryl-CoA. *J. Inherit. Metab. Dis.* **37**, 13–19 (2014). 23893049 10.1007/s10545-013-9632-0

[30] Little, A. S. et al. Dietary- and host-derived metabolites are used by diverse gut bacteria for anaerobic respiration. *Nat. Microbiol.* **9**, 55–69 (2024). 38177297 10.1038/s41564-023-01560-2 PMC11055453

[31] Mills, E. L. et al. Itaconate is an anti-inflammatory metabolite that activates Nrf2 via alkylation of KEAP1. *Nature* **556**, 113–117 (2018). 29590092 10.1038/nature25986 PMC6047741

[32] Bambouskova, M. et al. Itaconate confers tolerance to late NLRP3 inflammasome activation. *Cell Rep.* **34**, 108756 (2021). 33691097 10.1016/j.celrep.2021.108756 PMC8039864

[33] Hooftman, A. et al. The immunomodulatory metabolite itaconate modifies NLRP3 and inhibits inflammasome activation. *Cell Metab.* **32**, 468–478.e7 (2020). 32791101 10.1016/j.cmet.2020.07.016 PMC7422798

[34] Lampropoulou, V. et al. Itaconate links inhibition of succinate dehydrogenase with macrophage metabolic remodeling and regulation of inflammation. *Cell Metab.* **24**, 158–166 (2016). 27374498 10.1016/j.cmet.2016.06.004 PMC5108454

[35] Toma, I. et al. Succinate receptor GPR91 provides a direct link between high glucose levels and renin release in murine and rabbit kidney. *J. Clin. Invest.* **118**, 2526–2534 (2008). 18535668 10.1172/JCI33293 PMC2413183

[36] Lei, W. et al. Activation of intestinal tuft cell-expressed Sucnr1 triggers type 2 immunity in the mouse small intestine. *Proc. Natl Acad. Sci. USA* **115**, 5552–5557 (2018). 29735652 10.1073/pnas.1720758115 PMC6003470

[37] Best, N. et al. Bile acids drive the newborn’s gut microbiota maturation. *Nat. Commun.* **11**, 3692 (2020). 32703946 10.1038/s41467-020-17183-8 PMC7378201

[38] Tian, Y. et al. The microbiome modulating activity of bile acids. *Gut Microbes* **11**, 979–996 (2020). 32138583 10.1080/19490976.2020.1732268 PMC7524280

[39] Chen, M. et al. Itaconate is an effector of a Rab GTPase cell-autonomous host defense pathway against *Salmonella*. *Science* **369**, 450–455 (2020). 32703879 10.1126/science.aaz1333 PMC8020367

[40] Millet, Y. A. et al. Insights into *Vibrio cholerae* intestinal colonization from monitoring fluorescently labeled bacteria. *PLoS Pathog.* **10**, e1004405 (2014). 25275396 10.1371/journal.ppat.1004405 PMC4183697

[41] Kwai, B. X. C. et al. Itaconate is a covalent inhibitor of the *Mycobacterium tuberculosis* isocitrate lyase. *RSC Med. Chem.* **12**, 57–61 (2021). 34046597 10.1039/d0md00301h PMC8130629

[42] Rivera-Chávez, F. & Mekalanos, J. J. Cholera toxin promotes pathogen acquisition of host-derived nutrients. *Nature* **572**, 244–248 (2019). 31367037 10.1038/s41586-019-1453-3 PMC6727848

[43] Jouanna, J. in *Greek Medicine from Hippocrates to Galen* (ed. van der Eijk, P.) Ch. 16 335–360 (Brill, 2012).

[44] Zhu, S. et al. Nlrp9b inflammasome restricts rotavirus infection in intestinal epithelial cells. *Nature* **546**, 667–670 (2017). 28636595 10.1038/nature22967 PMC5787375

[45] Mohanty, I. et al. The underappreciated diversity of bile acid modifications. *Cell* **187**, 1801–1818.e20 (2024). 38471500 10.1016/j.cell.2024.02.019 PMC12248420

[46] Campbell, I. W., Hullahalli, K., Turner, J. R. & Waldor, M. K. Quantitative dose–response analysis untangles host bottlenecks to enteric infection. *Nat. Commun.* **14**, 456 (2023). 36709326 10.1038/s41467-023-36162-3 PMC9884216

[47] Ford, L. et al. Precision of a clinical metabolomics profiling platform for use in the identification of inborn errors of metabolism. *J. Appl. Lab. Med.* **5**, 342–356 (2020). 32445384 10.1093/jalm/jfz026

[48] Yuan, M., Breitkopf, S. B., Yang, X. & Asara, J. M. A positive/negative ion-switching, targeted mass spectrometry-based metabolomics platform for bodily fluids, cells, and fresh and fixed tissue. *Nat. Protoc.* **7**, 872–881 (2012). 22498707 10.1038/nprot.2012.024 PMC3685491

[49] Connolly, J. P. R. et al. Host-associated niche metabolism controls enteric infection through fine-tuning the regulation of type 3 secretion. *Nat. Commun.* **9**, 4187 (2018). 30305622 10.1038/s41467-018-06701-4 PMC6180029

[50] Costea, P. I. et al. Towards standards for human fecal sample processing in metagenomic studies. *Nat. Biotechnol.* **35**, 1069–1076 (2017). 28967887 10.1038/nbt.3960

[51] Bolyen, E. et al. Reproducible, interactive, scalable and extensible microbiome data science using QIIME 2. *Nat. Biotechnol.* **37**, 852–857 (2019). 31341288 10.1038/s41587-019-0209-9 PMC7015180

[52] Lin, H. & Peddada, S. D. Analysis of compositions of microbiomes with bias correction. *Nat. Commun.* **11**, 3514 (2020). 32665548 10.1038/s41467-020-17041-7 PMC7360769

[53] Beghini, F. et al. Integrating taxonomic, functional, and strain-level profiling of diverse microbial communities with bioBakery 3. *eLife* **10**, e65088 (2021). 33944776 10.7554/eLife.65088 PMC8096432

[54] Wood, D. E., Lu, J. & Langmead, B. Improved metagenomic analysis with Kraken 2. *Genome Biol.* **20**, 257 (2019). 31779668 10.1186/s13059-019-1891-0 PMC6883579

[55] Beresford-Jones, B. S. et al. The Mouse Gastrointestinal Bacteria Catalogue enables translation between the mouse and human gut microbiotas via functional mapping. *Cell Host Microbe*10.1016/j.chom.2021.12.003 (2021). 34971560 10.1016/j.chom.2021.12.003 PMC8763404

[56] Lu, J., Breitwieser, F. P., Thielen, P. & Salzberg, S. L. Bracken: estimating species abundance in metagenomics data. *PeerJ Comput. Sci.* **3**, e104 (2017). 10.7717/peerj-cs.104 PMC12016282 40271438

[57] Sit, B., Fakoya, B., Zhang, T., Billings, G. & Waldor, M. K. Dissecting serotype-specific contributions to live oral cholera vaccine efficacy. *Proc. Natl Acad. Sci. USA* **118**, e2018032118 (2021). 33558237 10.1073/pnas.2018032118 PMC7896348

[58] Rauckhorst, A. J. et al. Mouse tissue harvest-induced hypoxia rapidly alters the in vivo metabolome, between-genotype metabolite level differences, and 13C-tracing enrichments. *Mol. Metab.* **66**, 101596 (2022). 36100179 10.1016/j.molmet.2022.101596 PMC9589196

[59] Ferreyra, J. A. et al. Gut microbiota-produced succinate promotes *C. difficile* infection after antibiotic treatment or motility disturbance. *Cell Host Microbe* **16**, 770–777 (2014). 25498344 10.1016/j.chom.2014.11.003 PMC4859344

