# Supplementary material for: Enteric bacterial infection stimulates remodelling of bile metabolites to promote intestinal homeostasis
Source: Nat Microbiol. 2024 Nov 20;9(12):3376–90. doi: 10.1038/s41564-024-01862-z (PMC11602723; doi:10.1038/s41564-024-01862-z)
Supplement: Supplementary file 1 — Reporting Summary [file 41564_2024_1862_MOESM1_ESM.pdf]

nature portfolio

Corresponding author(s): Matthew Waldor

Last updated by author(s): Sep 20, 2024

# Reporting Summary

Nature Portfolio wishes to improve the reproducibility of the work that we publish. This form provides structure for consistency and transparency in reporting. For further information on Nature Portfolio policies, see our Editorial Policies and the Editorial Policy Checklist.

## Statistics

For all statistical analyses, confirm that the following items are present in the figure legend, table legend, main text, or Methods section.

| n/a | Confirmed | |
|---|---|---|
| ☐ | ☒ | The exact sample size (*n*) for each experimental group/condition, given as a discrete number and unit of measurement |
| ☐ | ☒ | A statement on whether measurements were taken from distinct samples or whether the same sample was measured repeatedly |
| ☐ | ☒ | The statistical test(s) used AND whether they are one- or two-sided<br>*Only common tests should be described solely by name; describe more complex techniques in the Methods section.* |
| ☒ | ☐ | A description of all covariates tested |
| ☐ | ☒ | A description of any assumptions or corrections, such as tests of normality and adjustment for multiple comparisons |
| ☐ | ☒ | A full description of the statistical parameters including central tendency (e.g. means) or other basic estimates (e.g. regression coefficient) AND variation (e.g. standard deviation) or associated estimates of uncertainty (e.g. confidence intervals) |
| ☐ | ☒ | For null hypothesis testing, the test statistic (e.g. *F*, *t*, *r*) with confidence intervals, effect sizes, degrees of freedom and *P* value noted<br>*Give P values as exact values whenever suitable.* |
| ☒ | ☐ | For Bayesian analysis, information on the choice of priors and Markov chain Monte Carlo settings |
| ☒ | ☐ | For hierarchical and complex designs, identification of the appropriate level for tests and full reporting of outcomes |
| ☒ | ☐ | Estimates of effect sizes (e.g. Cohen's *d*, Pearson's *r*), indicating how they were calculated |

*Our web collection on statistics for biologists contains articles on many of the points above.*

## Software and code

Policy information about availability of computer code

| | |
|---|---|
| Data collection | For liver RNA-sequencing, FASTQ files was generated using an Illumina NextSeq 550 instrument and Illumina's FASTQ generation pipeline (V1.0.0).<br>For 16S rRNA analysis, data was collected using an Illumina Mi-Seq instrument and FASTQ files were generated as described above. |
| Data analysis | For RNA-seq, low quality bases and the adaptors were trimmed by Trim Galore (v0.6.6) with a paired option. The reads were mapped to the mouse reference genome (mm10) using STAR v2.7.3a with default parameters, and gene-level raw reads count matrix was obtained by featureCounts function in the subread (v2.0.0). Differentially expressed genes were identified using the R-package DESeq2 (v1.36.0) with adjusted p-value < 0.05, absolute fold change of 1, and the clustering was performed using pam (Partitioning Around Medoids) function in the R-package cluster (v2.1.4) with a parameter 'k=7'. Pathway analysis was performed by the R-package clusterProfiler (v4.4.4) with gene sets from msigdbr R-package (v7.5.1) or KEGG metabolism pathways.<br>For 16S rRNA analysis, the Qiime2 pipeline was used to process the reads and the Greengene reference library was used for taxonomy mapping. Differential abundance of OTUs was analyzed using R package ANCOMBC.<br>For shotgun metagenomic sequencing, data were processed for quality control using KneadData. For taxonomical profiling, reads were processed using a k-mer method in Kraken2 (vKraken2) using a Kraken 2 database from The Mouse Gastrointestinal Bacterial Catalogue (MGBC) project. Taxonomical abundance was estimated using Bracken. Microbial functional profiling was performed using Humann3.<br>Statistical analysis was performed in Prism (v9.4.1).<br>For quantification of dicarboxylates abundance, samples were analyzed with using a 6500 QTRAP LC-MS/MS system and data were integrated using MultiQuant 3.0 for quantification across the sample set. |

For manuscripts utilizing custom algorithms or software that are central to the research but not yet described in published literature, software must be made available to editors and reviewers. We strongly encourage code deposition in a community repository (e.g. GitHub). See the Nature Portfolio guidelines for submitting code & software for further information.

## Data

Policy information about availability of data

All manuscripts must include a data availability statement. This statement should provide the following information, where applicable:

- Accession codes, unique identifiers, or web links for publicly available datasets
- A description of any restrictions on data availability
- For clinical datasets or third party data, please ensure that the statement adheres to our policy

Codes used for this paper are available at GitHub page: https://github.com/yh766/NMICROBIOL_Bile_2024 .Raw sequencing reads for liver transcriptome analysis are available at Gene Expression Omnibus (GEO accession number GSE227180). Raw sequencing reads for 16S and shotgun metagenomics analysis are available at the Sequencing Read Archive: (SRA accession number PRJNA947233).

## Research involving human participants, their data, or biological material

Policy information about studies with human participants or human data. See also policy information about sex, gender (identity/presentation), and sexual orientation and race, ethnicity and racism.

| | |
|---|---|
| Reporting on sex and gender | NA |
| Reporting on race, ethnicity, or other socially relevant groupings | NA |
| Population characteristics | NA |
| Recruitment | NA |
| Ethics oversight | NA |

Note that full information on the approval of the study protocol must also be provided in the manuscript.

# Field-specific reporting

Please select the one below that is the best fit for your research. If you are not sure, read the appropriate sections before making your selection.

☒ Life sciences ☐ Behavioural & social sciences ☐ Ecological, evolutionary & environmental sciences

For a reference copy of the document with all sections, see nature.com/documents/nr-reporting-summary-flat.pdf

# Life sciences study design

All studies must disclose on these points even when the disclosure is negative.

| | |
|---|---|
| Sample size | Sample sizes were determinated based on results of preliminary experiments. For untargeted metabolomics analysis, the samples we used are similar to previous study (PMID: 36100179). |
| Data exclusions | No data were excluded from the analyses. |
| Replication | The number of replication for each experiment were provided in figure legends. Key findings from global metabolic profiling were validated using independent methods. |
| Randomization | Animals were assigned randomly to experimental groups. |
| Blinding | Blinding were used for V. cholerae intestinal colonization assay and pathology scoring of H&E strained tissue slides. For the rest of experiments, most values were generated by sequencing, which is independent of investigator. |

# Reporting for specific materials, systems and methods

We require information from authors about some types of materials, experimental systems and methods used in many studies. Here, indicate whether each material, system or method listed is relevant to your study. If you are not sure if a list item applies to your research, read the appropriate section before selecting a response.

## Materials & experimental systems

| n/a | Involved in the study | |
|---|---|---|
| ☐ | ☒ | Antibodies |
| ☒ | ☐ | Eukaryotic cell lines |
| ☒ | ☐ | Palaeontology and archaeology |
| ☐ | ☒ | Animals and other organisms |
| ☒ | ☐ | Clinical data |
| ☒ | ☐ | Dual use research of concern |
| ☒ | ☐ | Plants |

## Methods

| n/a | Involved in the study | |
|---|---|---|
| ☒ | ☐ | ChIP-seq |
| ☒ | ☐ | Flow cytometry |
| ☒ | ☐ | MRI-based neuroimaging |

# Antibodies

| | |
|---|---|
| Antibodies used | Rabbit anti-DCAMK polyclonal antibody (ab31704, Abcam),<br>Alexa-594 Goat anti-Rabbit IgG secondary antibody (A-11072, Thermo Fisher),<br>rabbit anti-Acod1 polyclonal antibody (17805S, Cell signaling),<br>rabbit anti-HK2 polyclonal antibody (Proteintech, 22029-1-AP),<br>rabbit anti-GCDH polyclonal antibody (AV43559, Sigma),<br>peroxidase-conjugated goat anti-rabbit secondary antibody (A4914, Sigma).<br>Rabbit anti-CK19 monoclonal antibody (1:500 dilution, ab52625, Abcam) |
| Validation | All antibodies used for immunohistology and western blot analysis were purchased from Abcam, Sigma, Thermo Fisher, Cell signaling, and Proteintech. Validation information is provided by the manufacture's websites.<br>Rabbit anti-DCAMK polyclonal antibody (ab31704, Abcam, https://www.abcam.com/products/primary-antibodies/dcamkl1-antibody-ab31704.html ).<br>Alexa-594 Goat anti-Rabbit IgG secondary antibody (A-11072, Thermo Fisher, https://www.thermofisher.com/antibody/product/Goat-anti-Rabbit-IgG-H-L-Cross-Adsorbed-Secondary-Antibody-Polyclonal/A-11072).<br>rabbit anti-Acod1 polyclonal antibody (17805S, Cell signaling, https://www.cellsignal.com/products/primary-antibodies/irg1-antibody/17805).<br>rabbit anti-HK2 polyclonal antibody (Proteintech, 22029-1-AP, https://www.ptglab.com/products/HK2-Antibody-22029-1-AP.htm).<br>rabbit anti-GCDH polyclonal antibody (AV43559, Sigma, https://www.sigmaaldrich.com/US/en/product/sigma/av43559).<br>peroxidase-conjugated goat anti-rabbit secondary antibody (A4914, Sigma, https://www.sigmaaldrich.com/US/en/product/sigma/a4914).<br>Rabbit anti-CK19 monoclonal antibody (ab52625, Abcam, https://www.abcam.com/en-us/products/primary-antibodies/cytokeratin-19-antibody-ep1580y-cytoskeleton-marker-ab52625) |

# Animals and other research organisms

Policy information about studies involving animals; ARRIVE guidelines recommended for reporting animal research, and Sex and Gender in Research

| | |
|---|---|
| Laboratory animals | Special pathogen free (SPF) C57Bl/6J mice were purchased from the Jackson Laboratory (stock no. 000664). Germ free (GF) C57Bl/6J mice were purchased from the Massachusetts Host-Microbiome Center. Acod1-/- mice were purchased from the Jackson Laboratory (C57BL/6NJ-Acod1em1(IMPC)J/J, stock no. 029340) and bred in Harvard Medical School animal facility. Mice of 9-16 weeks of age were used for experiments. C57Bl/6 with 3-day postnatal infants (P3) were purchased from the Charles River Laboratories (stock no. 027) and kept in Harvard Medical School animal facility until postnatal day-5 (P5). All mice were kept under the 12-hour light-dark cycles: lights being turned off at 7 p.m. and turned on at 7 a.m. , with temperature (68-75F) and humidity (50%) controlled. Food and water were given ad libitum. |
| Wild animals | No wild animals were used in this study. |
| Reporting on sex | Female mice were used for bile metabolomic profiling, liver RNAseq, and fecal microbiota analysis. Male mice were used for tuft cell immunohistology. For V. cholerae intestinal colonization assay, both male and female infant mice (postnatal day-5, P5) were used. |
| Field-collected samples | No field-collected samples were used in this study. |
| Ethics oversight | All animal experiments were conducted following the protocol (2016N000416) reviewed and approved by the Brigham and Women's Hospital Institutional Animal Care and Use Committee. |

Note that full information on the approval of the study protocol must also be provided in the manuscript.

## Plants

| | |
|---|---|
| Seed stocks | NA |
| Novel plant genotypes | NA |
| Authentication | NA |
