# Supplementary figures and images for: Enteric bacterial infection stimulates remodelling of bile metabolites to promote intestinal homeostasis
Source: Nat Microbiol. 2024 Nov 20;9(12):3376–90. doi: 10.1038/s41564-024-01862-z (PMC11602723; doi:10.1038/s41564-024-01862-z)

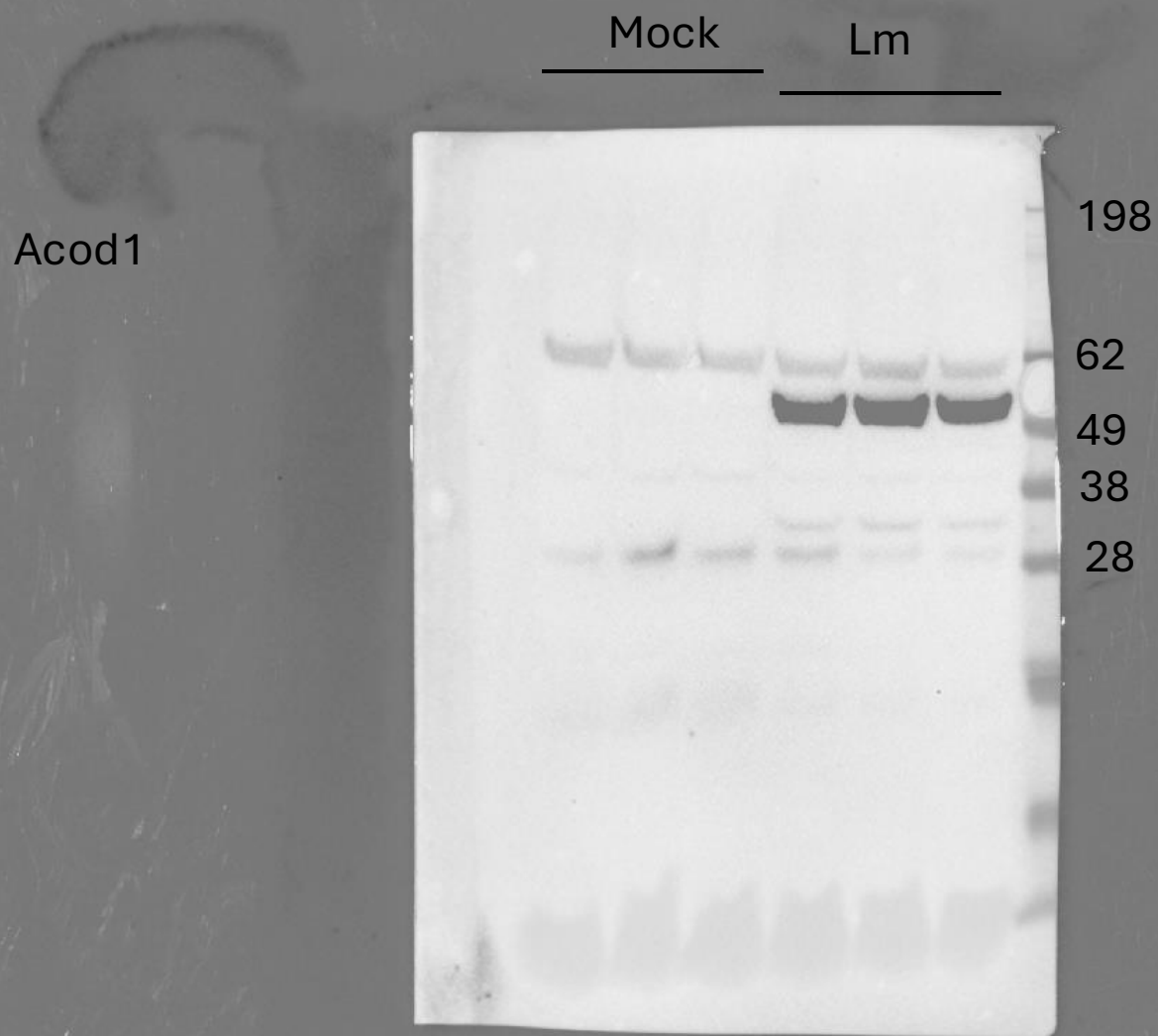

HK2

Mock

Lm

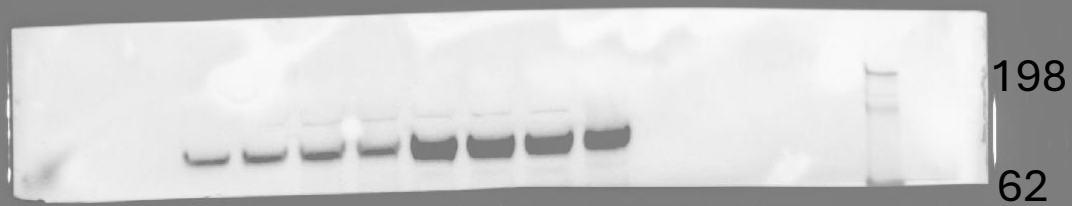

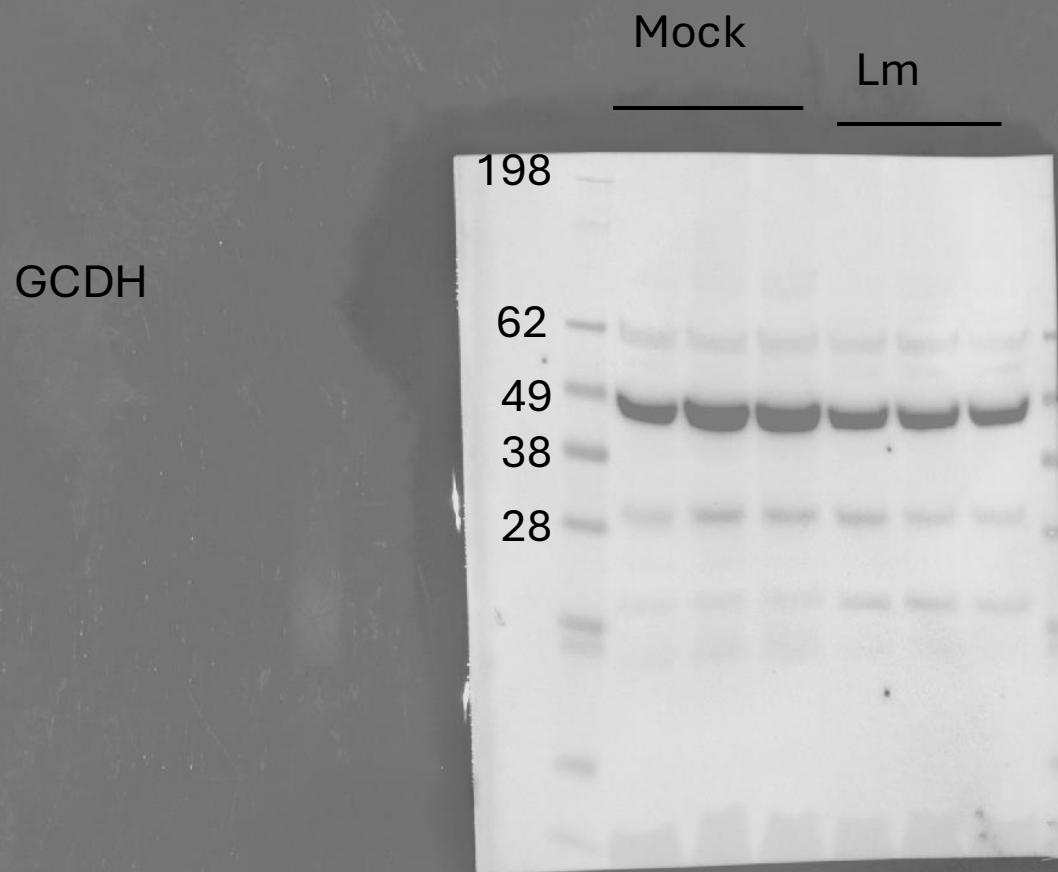

Ponceau S

Mock

Lm

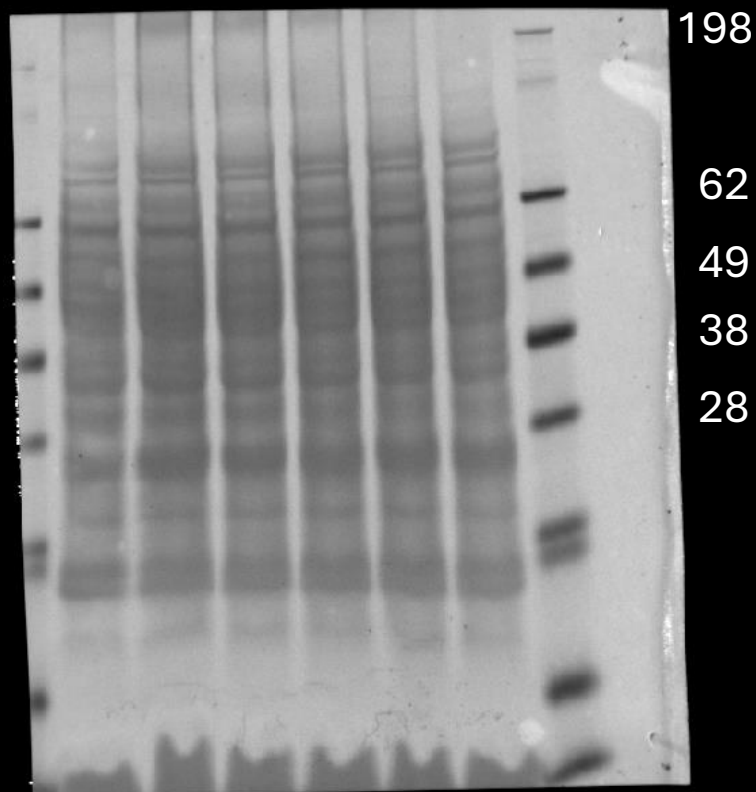

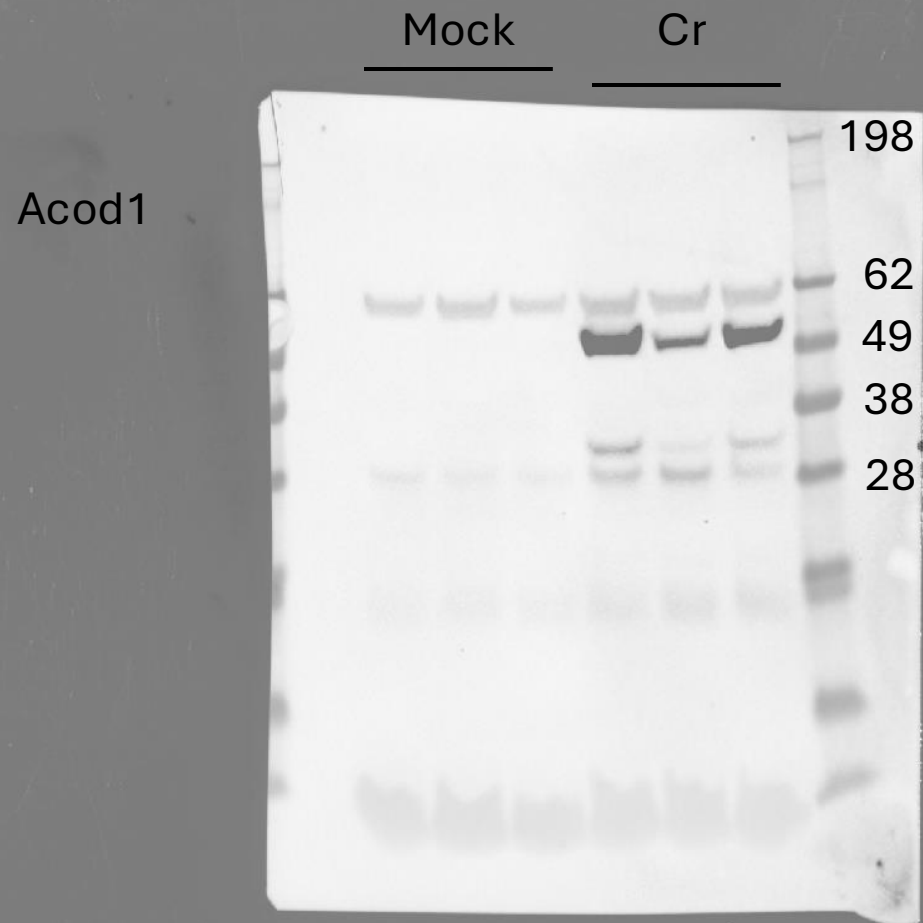

HK2

Mock

Cr

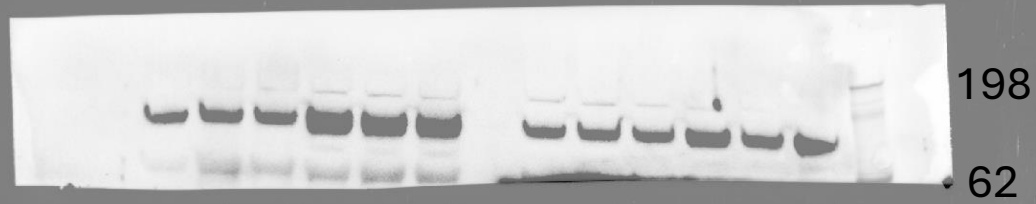

GCDH

Mock

Cr

198

62

49

38

28

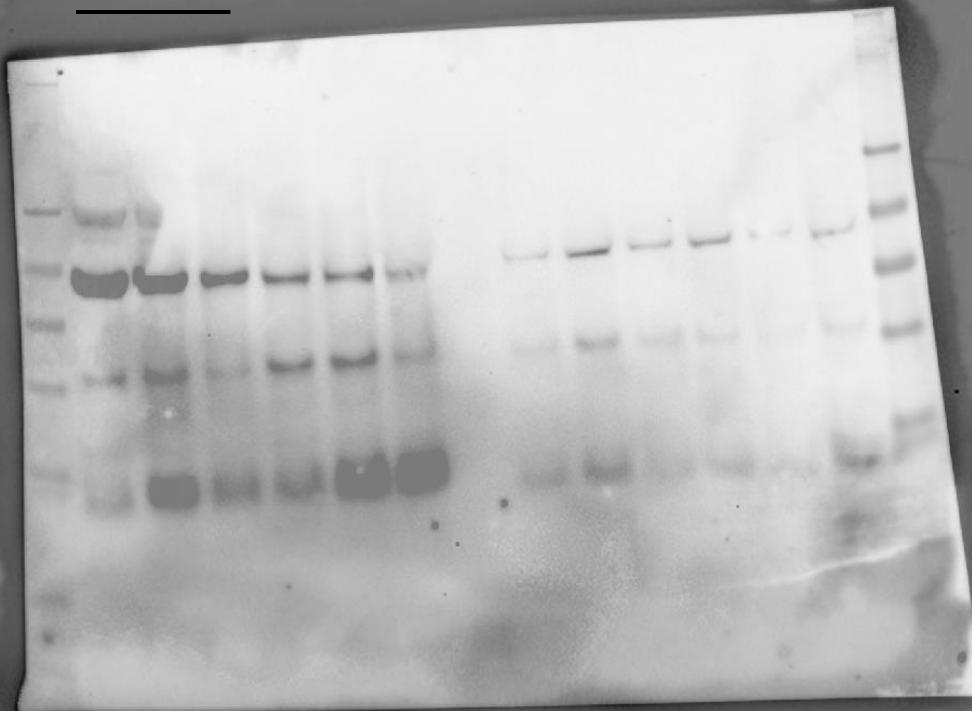

Ponceau S

Mock

Cr

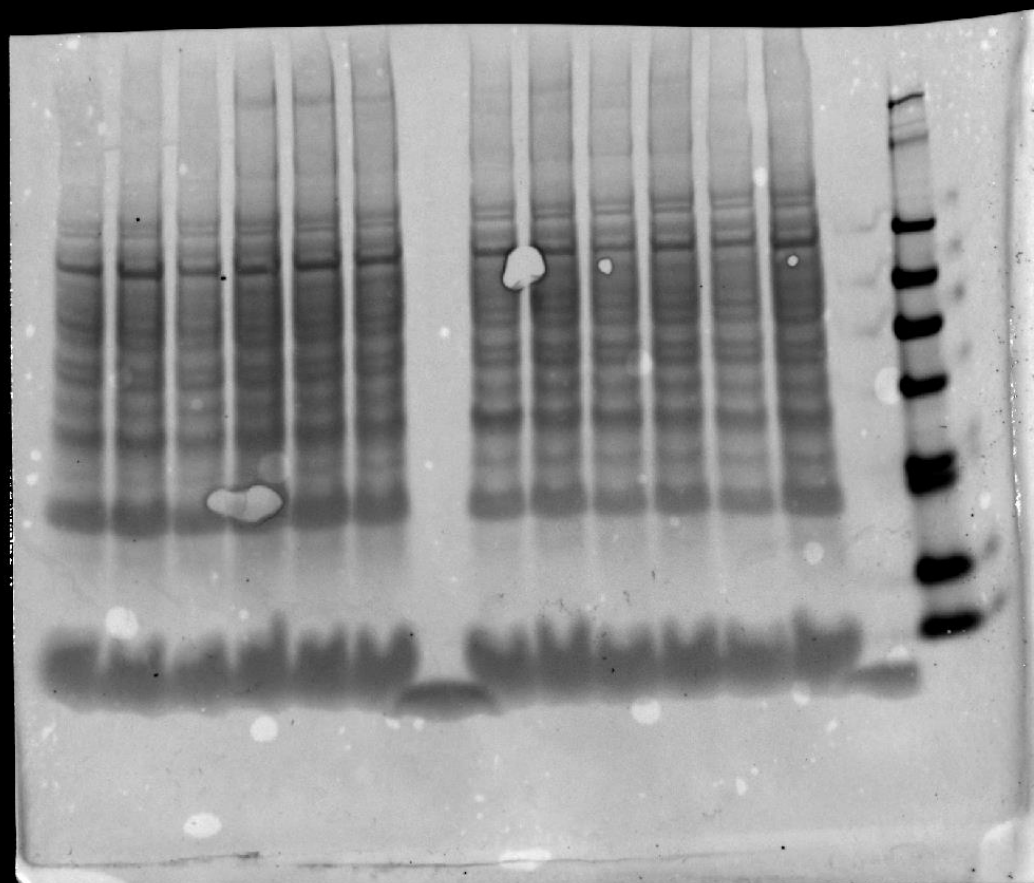

198

62

49

38

28

Supplement: Supplementary file 5 — Unprocessed western blots and/or gels. [file 41564_2024_1862_MOESM5_ESM.pdf]
